# A digital platform for the design of patient-centric supply chains

**DOI:** 10.1038/s41598-022-21290-5

**Published:** 2022-10-17

**Authors:** Niki Triantafyllou, Andrea Bernardi, Matthew Lakelin, Nilay Shah, Maria M. Papathanasiou

**Affiliations:** 1 Sargent Centre for Process System Engineering, Imperial College London, London, SW7 2AZ UK; 2 Department of Chemical Engineering, Imperial College London, London, SW7 2AZ UK; 3 TrakCel Limited, 10/11 Raleigh Walk, Cardiff, CF10 4LN UK

**Keywords:** Chemical engineering, Cancer immunotherapy

## Abstract

Chimeric Antigen Receptor (CAR) T cell therapies have received increasing attention, showing promising results in the treatment of acute lymphoblastic leukaemia and aggressive B cell lymphoma. Unlike typical cancer treatments, autologous CAR T cell therapies are patient-specific; this makes them a unique therapeutic to manufacture and distribute. In this work, we focus on the development of a computer modelling tool to assist the design and assessment of supply chain structures that can reliably and cost-efficiently deliver autologous CAR T cell therapies. We focus on four demand scales (200, 500, 1000 and 2000 patients annually) and we assess the tool’s capabilities with respect to the design of responsive supply chain candidate solutions while minimising cost.

## Introduction

**Figure 1:**
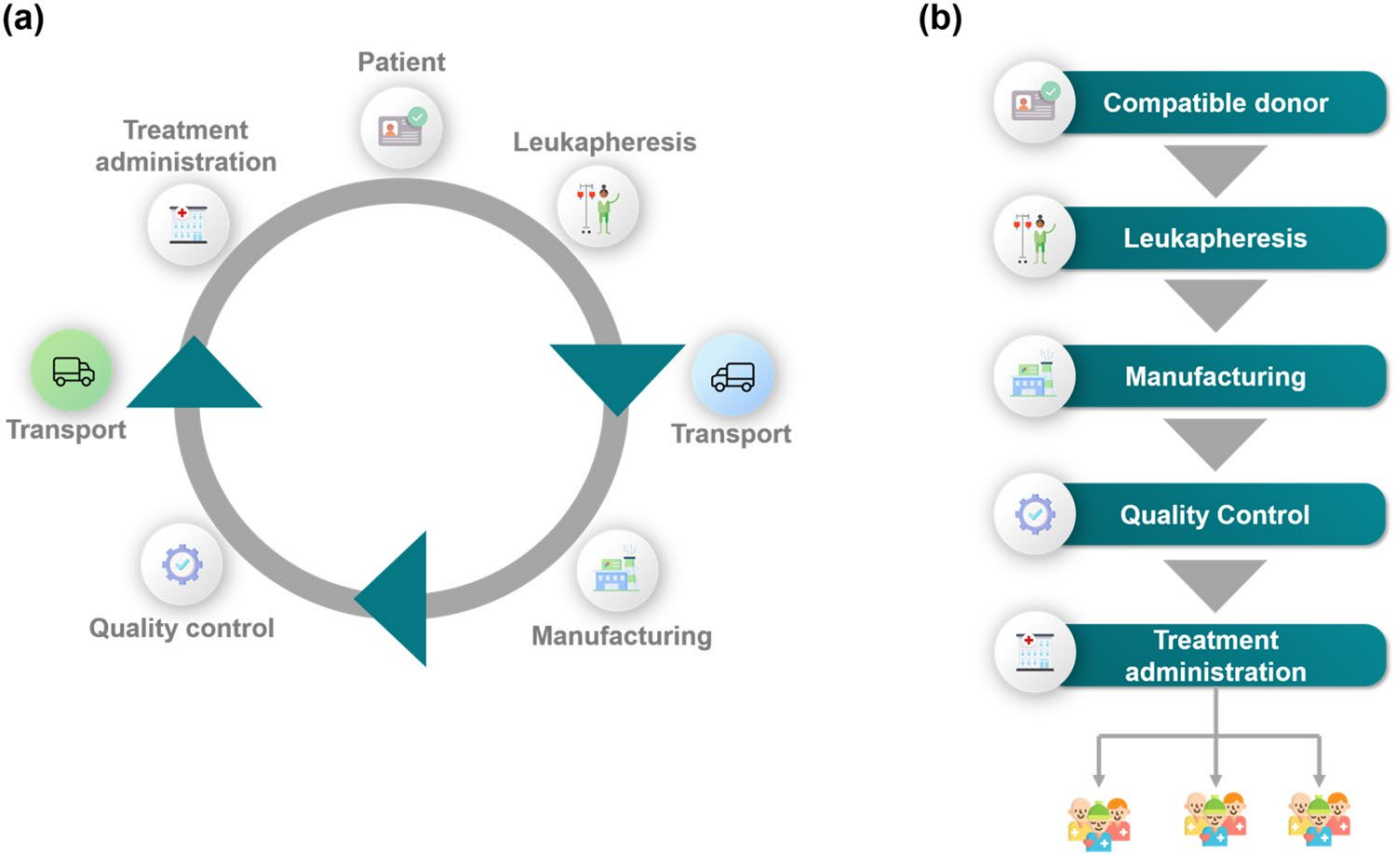
CAR T cell therapy lifecycle for (**a**) autologous and (**b**) allogeneic therapies.

Chimeric Antigen Receptor (CAR) T cell therapy is an emerging cancer treatment with promising results, particularly in relapsing B cell lymphoma and acute lymphoblastic leukaemia (ALL) patients^1–4^. In principle, the therapy is based on genetically engineering T cells to enable them to recognise and kill cancer cells^1,2^. These pioneering therapies can be either autologous or allogeneic, with the former considering the patient as the donor and the latter using the T cells of a healthy donor (Fig. 1). Allogeneic CAR T cells are still in clinical trials, tackling challenges arising from donor-to-donor variabilities^3^. On the other hand, autologous CAR T cells have received landmark approvals by the U.S. Food and Drug Administration (FDA) (2017) and European Medicines Agency (EMA) (2018)^4^ increasing hopes for a step-change in cancer treatment.

Novartis’ Kymriah, Gilead’s Yescarta and Tecartus, Bristol Myers Squibb’s Breyanzi and Abecma, and Janssen’s Carvykti are currently the six marketed autologous CAR T cell therapies offered at a relatively high list price, typically over $300,000 per therapy per patient^5–8^. They follow *pull-pull* supply chain strategies, where production and distribution planning are based on real-time patient orders. In contrast to batch-produced medicinal products, each individual autologous CAR T cell therapy is considered a separate product, requiring a patient-specific manufacturing and distribution schedule. This results in high manufacturing and distribution costs^9^. Despite their promising clinical results, CAR T cells remain a challenging therapeutic to manufacture and safely deliver at a larger scale^10^. Figure 1 illustrates the typical steps involved in the autologous CAR T cell therapy supply chain^11–14^. We have previously identified key operational and distribution challenges associated with the manufacturing and delivery of CAR T cells^15^. Further to that, we have designed a Resource Task Network (RTN) highlighting the main processing steps, which forms the basis of the work presented here.

CAR T cell manufacturing begins at the clinical site (hospital or specialist centre), where T cells are removed and isolated from the patient’s bloodstream. This procedure is known as leukapheresis. The isolated T cells are then shipped to the manufacturing facility, where they undergo a series of modifications including genetic engineering, expansion, and quality control^9^. Once the therapy is considered safe for the patient, it is released and shipped to the hospital for administration. Throughout the product lifecycle, samples can be transported either “fresh” (-80 °C) or “cryopreserved” (below-150 °C) depending on the manufacturer’s protocol^16^. Cryopreservation adds flexibility to the supply chain as it allows to extend the product shelf life, rendering it beneficial from both the patient and manufacturer perspective^17^.

The complete vein-to-vein procedure involves the coordination and availability of different raw materials, as well as expert handling during the transportation of the samples and/or therapies to the manufacturing facility for almost-immediate processing. At the same time, the final therapy needs to be carefully traced back to the original patient. This requires care in the formulation of the model for tractability while retaining this key feature, as well as the other features such as cycle times. Decisions related to the location of facilities, mode of transport and material stockpiling have to be taken in advance in order to ensure that the therapies will be manufactured safely and will be available on time^15^. At the same time, there is a pressing need to coordinate the manufacturing and distribution lifecycle with the patient schedule. The autologous nature of these therapies indicates patient-specific manufacturing batches, hindering volumetric scale-up and placing the patient schedule at the centre of the supply chain^18^. This dictates the coordination of therapy manufacturing and delivery based on the clinical condition and location of each patient separately.

The CAR T cell therapy lifecycle involves several transport and storage steps, which may expose the product to temperature excursions, risking its efficacy and safety^19^. Such events may become more likely in the case of white-glove logistics, which may not guarantee a responsive and resilient distribution network. On that front, digital tools can assist the supply chain design and optimisation orchestrating related processes and transition steps aiming to maintain therapy quality and minimise human error^19–21^. In this work, we harness the potential of Mixed Integer Programming (MIP) for the development of a comprehensive framework and digital platform to assist decision-making in investment planning and distribution of CAR T cell therapies. To our knowledge, this is the first tool that formally exploits the benefits of MIP for the identification of good candidate supply chains, by placing the patient in the centre of the decision-making.

In this work, we present i-SHIPMENT, a digital platform used for Individualised Supply cHain oPtimisation in Personalised MEdicine Treatments. The platform is based on a MILP model used for the identification of candidate supply chain structures and their operational details for safe and in-time delivery of CAR T cell therapies. The Key Performance Indicators (KPIs) considered here are the cost and the return time of the therapy to the patient. Cost is considered as the objective function, while “delivery time” is modelled as a novel non-monetary supply chain performance indicator expressed as a constraint. The model performance is assessed under four different demand scenarios (200, 500, 1000 and 2000 patients annually), assuming two different manufacturing durations (7 and 19 days). To reflect the current state of the art, we impose a constraint on the manufacturing facilities to be established (constrained scenarios). As a first step towards assessing a point-of-care manufacturing^22–24^, we assess a forward-looking scenario of decentralised manufacturing, where no constraint on the maximum number of manufacturing facilities is imposed (unconstrained scenarios).

### Mixed integer linear programming

**Figure 2:**
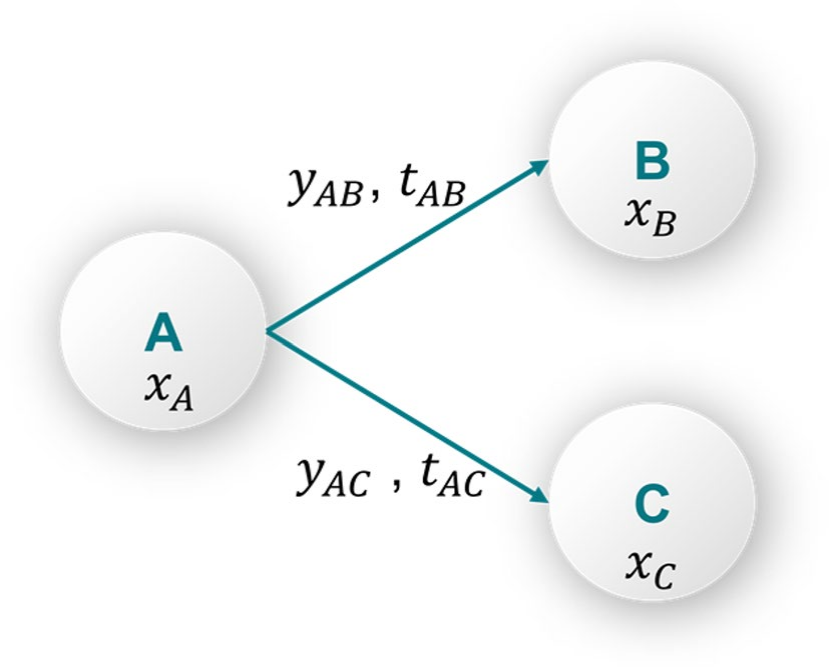
Explicit theoretical description of flow equations and binary decisions.

The mathematical formulation supporting i-SHIPMENT presented in the “Methods” section has been developed based on Mixed Integer Linear Programming (MILP). Figure 2 illustrates the basic concepts of MILP involving two types of variables, namely continuous ($$x\in {\mathbb{R}}^{n}$$) and binary $$(y\in {\{0, 1\}}^{m}).$$ Supply chain models that employ MIP, entail continuous variables that are used to describe the flow of materials and binary decisions related to the choice of facilities, routes, and transport modes. Assuming a supply chain with three nodes A, B and C (Fig. 2) we use continuous variables ($${x}_{A}, {x}_{B,}, {x}_{C}$$) to denote the flow of material $$x$$. We also use binary variables ($${y}_{AB}, {y}_{AC})$$ that take the value of 1 if the route is chosen and 0 otherwise. Lastly, $${t}_{AB}, {t}_{AC}$$ denote the time it takes to transport $$x$$ from node A to node B and from node A to node C respectively. Routes can either be mutually exclusive (when $${y}_{AB}=1$$ then $${y}_{AC}=0$$ and vice versa) or not.

Equation (1)–(7) present the journey of material ${\varvec{x}}$:

1 $$x_{A} = x_{{B + t_{AB} }} + x_{{C + t_{AC} }}$$

2 $$y_{AB} \cdot B^{L} \le x_{B} \le y_{AB} \cdot B^{U}$$

3 $$y_{AC} \cdot B^{L} \le x_{C} \le y_{AC} \cdot B^{U}$$

4 $$x_{A} , x_{B,} , x_{C} \in {\mathbb{R}}$$

5 $$y_{AB} , y_{AC} \in \left\{ {0, 1} \right\}$$

6 $$B^{L} , B^{U} \in {\mathbb{R}}$$

7 $$t_{AB} , t_{AC} \in {\mathbb{R}}$$

Equation (1) describes the flow of materials from node A to nodes B and C. In this relationship the time delay associated with transport durations ${t}_{AB}$ and ${t}_{AC}$ is considered. This ensures that the mathematical formulation will consider the sequence of events as they take place. Equations (2) and (3) ensure that there is a minimum and a maximum flow of materials between two nodes if a route is chosen, and it is equal to zero otherwise. This is described by multiplying the binary variables ${y}_{AB}, {y}_{AC}$ with a lower (${B}^{L}$) and upper (${B}^{U}$) bound value respectively. The upper and lower bounds are chosen by the modeller to best reflect the real-world application. Lastly, Eq. (4),(5) are the definitions of the continuous and binary variables. Supply chain optimisation models are aiming to identify the best possible value of a given Key Performance Indicator (KPI) (objective function) subject to a set of constraints. Both the objective function and the constraints are formulated based on the problem of interest. When all the equations in the set are linear then the problem is referred to as MILP.

MILP has been the backbone of supply chain optimisation problems developed for different application areas, including energy systems^25–34^, pharmaceuticals^35–45^ as well as food systems engineering^46–52^. The published tools are often based on modelling principles overarching the various formulations while being adapted to serve the specific network at hand^53–61^. Our proposed work is based on MILP principles and serves as the first holistic supply chain model in personalised cancer therapies. This is the first time that demand uncertainty, manufacturing capacity limitations, patient-specificity, tight time and location constraints are considered simultaneously. One of the main contributions of i-SHIPMENT is the central role that patients play in the decision-making process. This is translated into constraints on the total return time of the therapies to ensure that an upper bound is imposed, while the model is tracking each patient-therapy sample separately, hence ensuring that the 1:1 nature of the therapeutics is accurately captured. This is further explained in the Methodology.

## Results

Our results are presented in five sections. The first section is dedicated to the description of the digital platform i-SHIPMENT. i-SHIPMENT is used for the identification of good candidate supply chains, assessed based on two patient-centric KPIs; namely the average cost per therapy and therapy return time. At the same time, we consider key decisions that manufacturers need to make to ensure safe delivery, sufficient capacity to fulfil the demand and responsiveness to the patient needs. Such decisions may include the number and capacity of the manufacturing facilities, as well as their location. Here, we assess the current state-of-the-art in manufacturing (19 days) and a forward-looking scenario (7 days). Based on industrial practice, we set an upper limit on the average return time to ensure that the generated solutions fulfil patient needs. i-SHIPMENT identifies the optimal number and location of the manufacturing facilities to be established, transport modes for the node-to-node connections, scheduling of the therapies in the manufacturing sites and hospitals, as well as the utilisation of the manufacturing sites with the paramount objective of minimising the therapy cost and return time. In the following, we present four case studies of different demand profiles (200, 500, 1000 and 2000 patients annually) (Figs. 4–7). All the information on the locations and data considered in this work can be found in Supplementary Tables 1–4. Supplementary Table 10 summarises all the key results and Supplementary Figs. 1–7 display the % utilisation of manufacturing facilities for all scenarios.

### i-SHIPMENT: the digital platform

**Figure 3:**
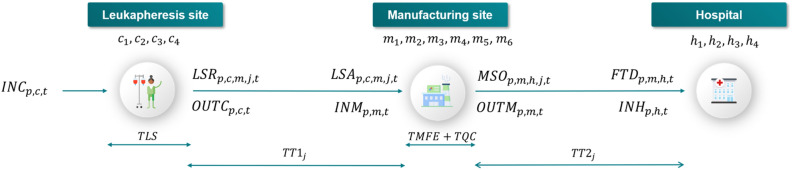
CAR T cell therapy lifecycle as considered in this work. $INC_{p,c,t}$ corresponds to the incoming patient $p$ at the leukapheresis site $c$ at time $t$. The outgoing leukapheresis sample is ready to be shipped to the manufacturing facility $m$ after $TLS$ which is the duration of the leukapheresis procedure. At the leukapheresis site exit we consider two variables, namely: $OUTC_{p,c,t}$ and $LSR_{p,c,m,j,t}$ both indicating the leukapheresis samples of patient $p$, ready to be shipped from the leukapheresis site $c$ at time $t$. Their main difference is that the former ($OUTC_{p,c,t}$) allows tracking of the general mass balance around the facility, while $LSR_{p,c,m,j,t}$ enables tracking of the leukapheresis sample of patient $p$, ready to be shipped from the leukapheresis site $c$ to the manufacturing facility $m$, using transport mode $j$ at time $t$. $TT1_{j}$ corresponds to the transportation duration between the leukapheresis site and the manufacturing facility based on mode $j$. After $TT1_{j}$ the leukapheresis samples arrive at the manufacturing facility $m$. In the same fashion, we consider two variables both at the entrance and at the exit of the manufacturing facility. $INM_{p,m,t}$ and $OUTM_{p,m,t}$ are used to describe the general mass balance between incoming and outgoing therapies of patient $p$ to and from the manufacturing facility $m$, respectively, at time $t$. Similarly, $LSA_{p,c,m,j,t}$ enables tracking of the incoming therapies $p$ from leukapheresis site $c$ to manufacturing facility $m$ using transport mode $j$ at time $t$. $MSO_{p,m,h,j,t}$ describes the outgoing therapies $p$ from manufacturing facility $m$ to hospital site $h$ using transport mode $j$ at time $t$. Therapies will be ready to leave the manufacturing after $TMFE + TQC$ which is the sum of the manufacturing and quality control durations. After $TT2_{j}$ the manufactured therapies arrive at the hospital site $h$. In the same way, we use two variables to denote the incoming therapies. Specifically, $INH_{p,h,t}$ that describes the general flow of therapies $p$ arriving at the hospital site $h$ at time $t$ and $FTD_{p,m,h,j,t}$ that tracks therapy $p$ that has left from manufacturing facility $m$ and is arriving at the hospital site $h$ via transport mode $j$ at time $t$.

i-SHIPMENT is a digital platform based on a set of linear equations, using both continuous and binary variables. The mathematical model supporting i-SHIPMENT considers the supply chain structure as illustrated in Fig. 3, whereby therapy manufacturing starts with the leukapheresis procedure at a clinical site and/or hospital. i-SHIPMENT aims to minimise the total cost ($TOTCOST$), while satisfying a series of constraints as presented in the “Methods” section. The differentiation in the granularity of the variable indexing enables close tracking of the sample journey and its association with the chosen transport mode.

### Case 1: 200 patients/year

**Figure 4:**
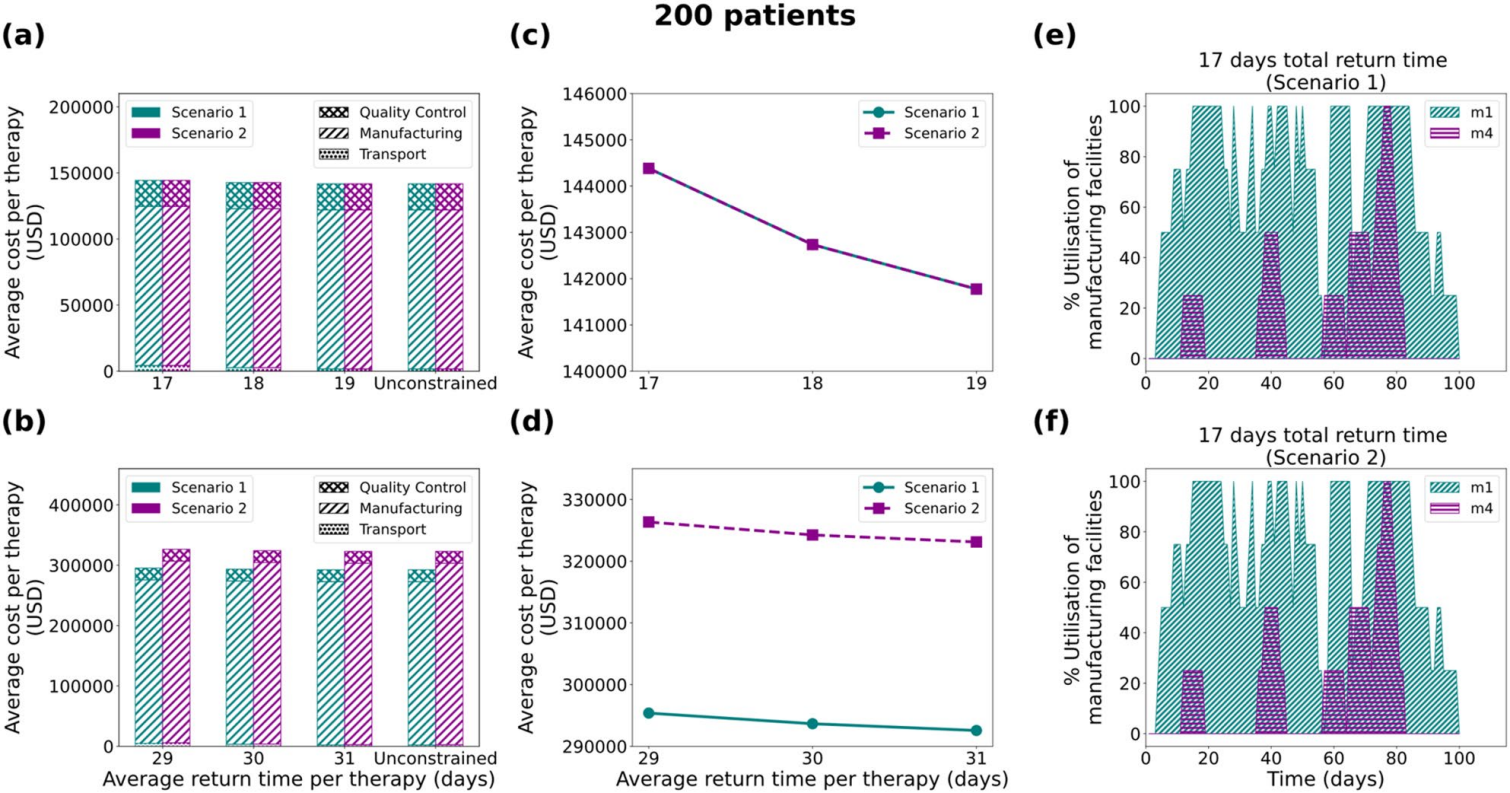
Results for the 200 patients/year demand scenario: Comparison of average cost per therapy (USD) for (**a**) 7 and (**b**) 19 days duration of the manufacturing process, where the cost is broken down into transport cost, manufacturing cost and Quality Control cost. Average cost per therapy as a function of the average return time for (**c**) 7 and (**d**) 19 days. Utilisation of manufacturing facilities built for the 7 days manufacturing duration for Scenario 1 (**e**) and Scenario 2 (**f**), with the average return time of therapy constrained at 17 days. Scenario 1 and Scenario 2 correspond to unconstrained and constrained number of manufacturing facilities respectively.

Figure 4 illustrates the results for all scenarios regarding the 200 patients/year case. It is observed that in both scenarios, the average cost per therapy decreases as a function of the vein-to-vein duration (Figs. 4c and 4d). For the 7 days duration of the manufacturing process, all cases for both scenarios (constrained and unconstrained number of facilities) result in the same average cost per therapy. On the other hand, the 19-day manufacturing process presents significant differences between the constrained (up to two manufacturing facilities) and the unconstrained case. It is observed that for the unconstrained case the model suggests the development and utilisation of three manufacturing facilities (*m*_*1*_, *m*_*3*_ and *m*_*4*_) with a total capacity of 18 therapies/week while for the constrained case only two facilities are built (*m*_*3*_ and *m*_*6*_) which results in a total capacity of 20 therapies/week (Supplementary Fig. 1). Here the counterintuitive outcome of the cost being inversely proportional to the number of facilities built can be attributed to the utilisation percentage of the respective facilities. For the 7 days manufacturing process, it can be seen (Figs. 4e and 4f) that for the constrained and unconstrained scenarios the optimisation model suggests investing in and using two manufacturing facilities (*m*_*1*_ and *m*_*4*_) with a total weekly capacity of 8 therapies.

### Case 2: 500 patients/year

**Figure 5:**
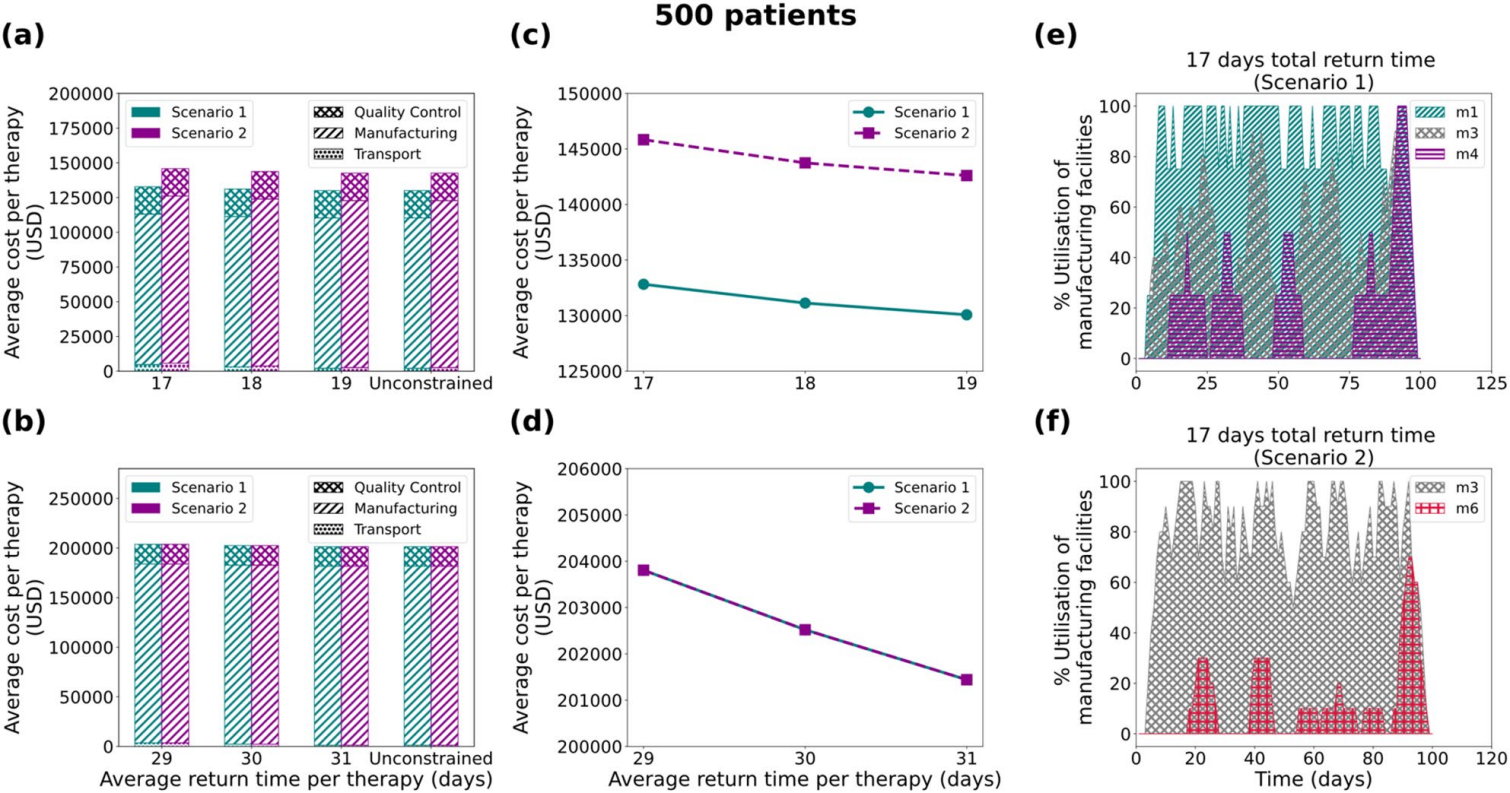
Results for the 500 patients/year demand scenario: Comparison of average cost per therapy (USD) for (**a**) 7 and (**b**) 19 days duration of the manufacturing process, where the cost is broken down into transport cost, manufacturing cost and Quality Control cost. Average cost per therapy as a function of the average return time for (**c**) 7 and (**d**) 19 days. Utilisation of manufacturing facilities built for the 7 days manufacturing duration for Scenario 1 (**e**) and Scenario 2 (**f**), with the average return time of therapy constrained at 17 days. Scenario 1 and Scenario 2 correspond to unconstrained and constrained number of manufacturing facilities respectively.

In the 500 patients/year case (Fig. 5) the 19-day manufacturing process scenario results in identical solutions for the constrained and unconstrained cases (Fig. 5b), whilst differences are observed in the cost for the two cases in the 7-day scenario. Based on the 17-day average total return time scenario, it is observed that for the unconstrained case (Fig. 5e) the model proposes the establishment of three manufacturing facilities (*m*_*1*_, *m*_*3*_ and *m*_*4*_) with a total capacity of 18 therapies/week, while for the constrained case (Fig. 5f) only two facilities are built (*m*_*3*_ and *m*_*6*_) with a total capacity of 20 therapies/week. Intuitively, one would think that the latter would result in a lower cost of therapy, however, the opposite is observed (Fig. 5a and 5c). As illustrated in Fig. 5f, *m*_*6*_ is underutilised (< 40% for most of the time), while in the unconstrained case the model alternates between the three facilities, therefore increasing their average utilisation.

**Figure 6:**
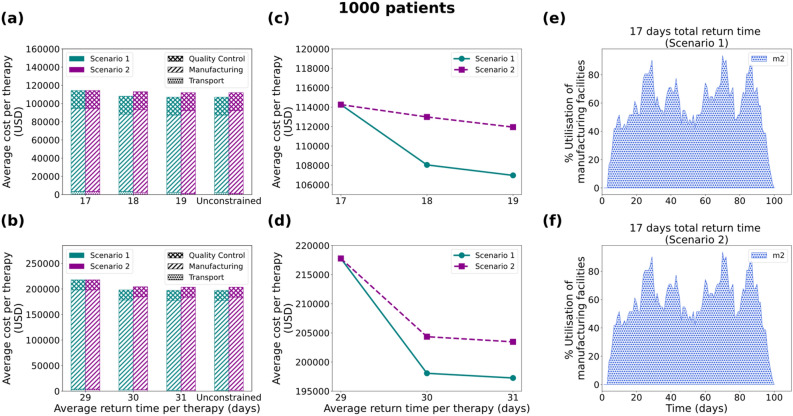
Results for the 1000 patients/year demand scenario: Comparison of average cost per therapy (USD) for (**a**) 7 and (**b**) 19 days duration of the manufacturing process, where the cost is broken down into transport cost, manufacturing cost and Quality Control cost. Average cost per therapy as a function of the average return time for (**c**) 7 and (**d**) 19 days. Utilisation of manufacturing facilities built for the 7 days manufacturing duration for Scenario 1 (**e**) and Scenario 2 (**f**), with the average return time of therapy constrained at 17 days. Scenario 1 and Scenario 2 correspond to unconstrained and constrained number of manufacturing facilities respectively.

### Case 3: 1000 patients/year

Next, the model is tested for 1000 patients/year (Fig. 6), where a similar behaviour is observed for both the 7-day and the 19-day duration of manufacturing. For most of the cases, both scenarios (constrained and unconstrained number of facilities) result in a different average cost of therapy, with two exceptions (Figs. 6a and 6b). The first one is the simulation assuming 7 days duration of the manufacturing process and an upper bound of 17 days for the return time per the therapy (Figs. 6a first column stack and 6c). Similarly, the 19-day manufacturing duration with an upper limit of 29 days for the return time of the therapy shows identical solutions between Scenario 1 and 2 (Fig. 6b, first column stack and 6d). As depicted in Figs. 6e and 6f, this is because for the 17-day total return time the model chooses to invest in and utilise only one manufacturing facility (*m*_*2*_) with weekly capacity of 31 therapies. In contrast, significant differences are observed in the other cases between the constrained and unconstrained number of facilities scenarios with the model suggesting different supply chain configurations (Supplementary Figs. 3 and 7).

### Case 4: 2000 patients/year

**Figure 7:**
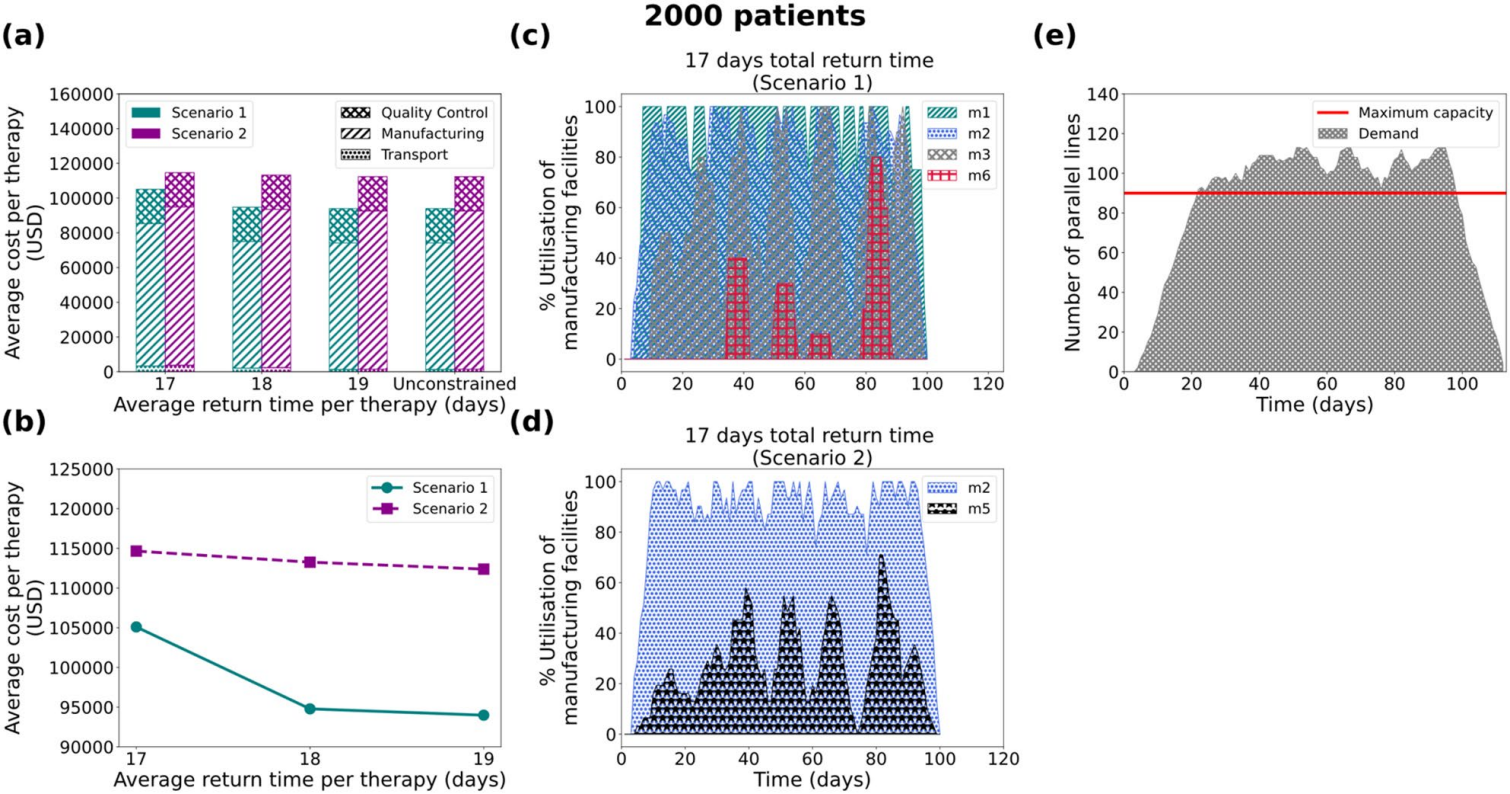
Results for the 2000 patients/year demand scenario: (**a**) Average cost per therapy (USD) for 7 days duration of the manufacturing process, where the cost is broken down into transport cost, manufacturing cost and Quality Control cost. (**b**) Average cost per therapy as a function of the average return time for 7 days of manufacturing. Utilisation of manufacturing facilities built for the 7 days manufacturing duration for Scenario 1 (**c**) and Scenario 2 (**d**), with the average return time of therapy constrained at 17 days. (**e**) Number of parallel lines needed for the 19-day manufacturing duration with the average return time of therapy being unconstrained. Scenario 1 and Scenario 2 correspond to unconstrained and constrained number of manufacturing facilities respectively.

For the 2000 patients/year case the model only provides solutions for the forward-looking scenario of 7 days manufacturing duration. Contrary to what was demonstrated previously for the 200-, 500- and 1000- patient cases, here, the 7-day manufacturing cases report an average optimality gap of 13.7% and 2.4% for Scenario 1 and Scenario 2, respectively. This is translated into the relative difference between the best bound found thus far and the optimal solution reported. Based on expert interviews we consider an uncertainty of up to 20% in the cost data as assumed in this work. This is primarily attributed to the reported market variability in the selling prices^62,63^ and the developing nature of the technologies involved in the manufacturing of CAR T cells. Therefore, the reported optimality gap can be considered representative of this uncertainty. The solutions for the 7-day duration are significantly different for Scenarios 1 and 2. It is observed that in the 7-day manufacturing process cases (Figs. 7a and 7b) the constrained solution with fewer manufacturing facilities results in a higher average cost per therapy. Taking the 17-day return time of therapy as an example, it is observed that in the constrained case (Scenario 2) the model suggests (Fig. 7d) building two manufacturing facilities (*m*_*2*_ and *m*_*5*_) with a weekly capacity of 62 therapies. In the unconstrained case (Scenario 1), the optimal solution suggests investing in and building four candidate manufacturing facilities (*m*_*1*_, *m*_*2*_, *m*_*3*_ and *m*_*6*_) that add up to a weekly capacity of 55 therapies. In this case, it is evident that the utilisation percentage of the facilities plays a more dominant role than the number of facilities used. In the unconstrained case (Fig. 7c), facilities are used rotationally and three of them reach maximum capacity at some time during the presented trimester. On the other hand, in the constrained case (Fig. 7d), facility *m*_*2*_ reaches full capacity at most time points, whereas facility *m*_*5*_ is significantly underutilised and operates at an average of 27% capacity. In the 19-day manufacturing scenario, the total capacity of the currently available manufacturing sites collectively is not sufficient to meet the demand. It is apparent in Fig. 7e that the maximum number of production parallel lines in the available manufacturing sites, even if all six candidate sites are built, is notably exceeded in most time points. This leads to an infeasible solution as the capacity constraints in the model cannot be satisfied.

## Discussion

In this study, we present a mathematical tool that can assist the decision-making process in the design of personalised healthcare supply chains. It is one of the first methodological attempts that quantifies cost-time trade-offs in this field while placing the patient in the centre of the decision-making process. The presented model (i-SHIPMENT) is used for the identification of good candidate solutions that guarantee manufacturing capacity, as well as responsiveness to patient needs.

### Variable cost as a function of the manufacturing utilisation percentage

As observed in the “Results” section, modelling variable manufacturing cost as a function of the utilisation percentage of the facility plays a dominant role in the design of the supply chain. As illustrated (Figs. 4e, 4f, 5e, 5f, 6e, 6f, 7c and 7d) the best candidate structure is not always the one involving fewer facilities, as one may intuitively think. On the contrary, there are cases where a decentralised manufacturing approach may result in more cost-efficient supply chains (Fig. 7c), even though most facilities are on average equally utilised. This can be a prelude to the investigation of decentralised supply chains for the production and distribution of personalised therapies that may reveal the potential of the point-of-care therapy model^10,14^. The scenarios illustrated here operate under a fixed total demand that results in a relatively constant average cost distribution (83.50% manufacturing, 4% transport and 12.5% QC). Nevertheless, as discussed in Papathanasiou et al.^15^, as CAR T cell therapies progress and may be offered to healthier subjects, the supply chain and associated logistics will also need to adapt. The latter is translated to agile supply chains that can tackle the increasing demand without changing their basic structure, but rather by utilising alternatives, such as intermediate storage to debottleneck the production lines.

### Model limitations

Here the demand is generated considering a random uniform distribution of patients with the only constraint of a maximum of 8 patients per day per leukapheresis centre (Please refer to the SI, Tables 7–9, Fig. 8). We assume that the demand profiles are the same across each trimester of the year. For the scenarios of 2000 patients and a 19-days manufacturing time, the total capacity of the candidate manufacturing facilities is not sufficient to meet the demand and therefore the model leads to infeasible solutions. Furthermore, the impact of the model complexity on the solution is also depicted in the 2000-patients results, where an optimality gap is reported. The latter is directly associated with the size of the model as it does not occur for the other three problems (200-, 500- and 1000- patient cases), where the formulation is smaller. To mitigate this, we are developing decomposition methods and an advanced algorithm that will be able to eliminate branches from the optimisation search space.

## Methods

### Cost-time trade-off

As demonstrated (Figs. 4c, 4d, 5c, 5d, 6c, 6d, 7b) the cost of therapy decreases as a function of the return time. This is mostly due to the choice of less expensive modes of transport when the time constraint is more relaxed. The integration of the return time as a constraint in the model formulation drives decisions related to the overall structure of the supply chain. For example, as illustrated in Figs. 5e and 5f, the model prioritises the use of facility *m*_*1*_ located in the UK in order to satisfy the strict time constraint ($TRT\le 17$), while decreasing the total cost. In this way, there is greater flexibility in choosing the fastest transport mode (24-h) delivery. The 1000-patient case differs in that respect as the capacity seems to be the main driver for the choice of facilities, thus the model shows no preference for locations that are closer to the patient. Such an example could demonstrate a different behaviour if there was higher distribution and variation amongst the candidate facilities with respect to location and capacity. Lastly, it is observed that in the 2000-patient case both time and capacity play an equal role in the prioritisation of the facilities as the model suggests *m*_*1*_ (UK), *m*_*2*_ (EU), *m*_*3*_ (EU) and *m*_*6*_ (UK) to be the first four to be utilised. It could be concluded that as patient demand increases, the dynamics of the supply chain vary and therefore cost categories that were otherwise not significant, become an integral part of the overall cost.

### Mathematical model of i-SHIPMENT

i-SHIPMENT assists decision-making in the CAR T cell therapies supply chain by identifying candidate structures of minimum cost that satisfy the indicated total return time of therapy constraint. The model has been developed based on the following assumptions:

- The leukapheresis sites are pre-defined, aligned with the UK healthcare system and the ATTCs
- Each therapy needs to return to the hospital that is co-located with the leukapheresis site from which the original sample was taken
- The capacity of the candidate manufacturing facilities is fixed
- The demand profile is repeated every trimester of the year

The objective of the model is to minimise the total cumulative cost of all manufactured therapies (Eq. 8).

8 $$\min TOTCOST = \mathop \sum \limits_{p} CTM_{p} + \mathop \sum \limits_{p} TTC_{p} + \mathop \sum \limits_{p} CQC$$

The total cost (Eq. 8) is calculated as the sum of the manufacturing ($$CTM_{p}$$), transport ($$TTC_{p}$$) and quality control costs ($$CQC)$$ for all patients $$p$$. In the scenarios presented here, quality control is considered an in-house procedure with a fixed equal cost for each patient. Supplementary Tables 1–4 include all the data as considered for the generation of the results presented here.

#### Manufacturing costs

 The first term of the objective function ($\mathop \sum \limits_{p} CTM_{p} )$ represents the manufacturing cost of all therapies $p$. Equation (9) calculates the manufacturing cost per therapy $p$.

9 $$CTM_{p} = \frac{{\mathop \sum \nolimits_{m} (E1_{m} \cdot (CIM_{m} + CFVM_{m} ))}}{NP} + CVM_{p} { },{ }\forall { }p$$

The first term of Eq. (9) ($\frac{{\mathop \sum \nolimits_{m} E1_{m} \cdot CIM_{m} }}{NP}$) corresponds to the capital investment for the construction of a new facility $m$ as attributed to each therapy $p$. The second term ($\frac{{\mathop \sum \nolimits_{m} E1_{m} \cdot CFVM_{m} }}{NP}$) reflects the fraction of variable manufacturing costs, also referred to as “fixed-variable costs”, that cannot be easily adjusted to variable demand once the target productivity has been decided. Those may include personnel, facilities and equipment maintenance costs. Such costs are ~ 80% of the total variable costs and are only dependent on the number of established parallel lines, regardless of the utilisation of the facility, therefore they can lead to a significant increase in the average cost per therapy if the average utilisation of a manufacturing facility is low^64^. The third term ($CVM_{p}$) describes the variable costs associated with the materials required for manufacturing. Given the autologous nature of the CAR T therapies, this cost is only marginally affected by economies of scale and we assume that it is independent of the size of the facility^64^.

Equation (10) calculates the percentage of utilisation of facility $m$ at time $t$.

10 $$RATIO_{m,t} = \frac{{\mathop \sum \nolimits_{p} DURM_{p,m,t} }}{{FCAP_{m} }},{ }\forall { }m,t$$

$DURM_{p,m,t}$ is a continuous variable that takes a value of 1 only for the time period $t$ in which a therapy $p$ is being manufactured in facility $m$.

#### Transport costs

 The second term in Eq. (8) ($\mathop \sum \limits_{p} TTC_{p}$) corresponds to the total cost of transport for all therapies $p$, while the transport cost per therapy $p$ ($TTC_{p}$) is given by Eq. (11). The two terms correspond to transport from: (1) clinical site to manufacturing site and (2) manufacturing site to hospital, respectively.

11 $$TTC_{p} = \mathop \sum \limits_{c,m,j,t} Y1_{p,c,m,j,t} \cdot TT1_{j} \cdot U1_{c,m,j} + \mathop \sum \limits_{m,h,j,t} Y2_{p,m,h,j,t} \cdot TT2_{j} \cdot U2_{m,h,j} ,{ }\forall { }p$$

In this work, we consider that QC is co-located with the manufacturing facility and therefore QC costs are fixed and provided in Supplementary Table 1.

#### Material balances

 The model formulation includes a series of material balances around the facilities that are described by Eq. (12)–(19). Patient samples $p$ collected at the leukapheresis site $c$ at time $t$ ($INC_{p,c,t}$) will be ready to be shipped to the manufacturing facility ($OUTC_{p,c,t}$) after the duration of the leukapheresis procedure ($TLS$):

12 $$INC_{p,c,t} = OUTC_{{p,c,t + TLS{ }}} ,{ }\forall p,c,t$$

Patient samples $p$ collected at the leukapheresis site $c$ being shipped to manufacturing facility $m$ via transport mode $j$ at time $t$ ($LSR_{p,c,m,j,t}$) will arrive at the manufacturing facility ($LSA_{p,c,m,j,t}$) after the duration of the transport activity ($TT1_{j}$) (Eq. 13):

13 $$LSR_{p,c,m,j,t} = LSA_{{p,c,m,j,t + TT1_{j} }} ,{ }\forall p,c,m,{ }j,t$$

For each patient sample $p$, the outgoing samples $OUTC_{p,c,t}$ from a leukapheresis site $c$ at time $t$ are equal to patient sample $p$ sent to all manufacturing facilities $m$ under any transport mode $j$ at time $t$ ($\mathop \sum \limits_{m,j} LSR_{p,c,m,j,t}$) (Eq. 14).

14 $$OUTC_{p,c,t} = \mathop \sum \limits_{m,j} LSR_{p,c,m,j,t} ,{ }\forall p,c,t$$

Patient sample $p$ entering manufacturing facility $m$ at time $t$ is equal to patient samples $p$ shipped from all leukapheresis sites $c$ to manufacturing facility $m$ under any transport mode $j$ ($\mathop \sum \limits_{c,j} LSA_{p,c,m,j,t}$) (Eq. 15).

15 $$INM_{p,m,t} = \mathop \sum \limits_{c,j} LSA_{p,c,m,j,t} ,{ }\forall p,m,t$$

The outlet of patient samples $$p$$ of a manufacturing facility $$m$$ at time $$t$$ ($$OUTM_{p,m,t}$$) will be ready for shipment after the manufacturing process ($$TMFE$$) and quality control ($$TQC)$$ have been completed (Eq. 16).

16 $$INM_{p,m,t} = OUTM_{p,m,t + TMFE + TQC} ,{ }\forall p,m,t$$

The sample $p$ leaving the manufacturing facility $m$, $OUTM_{p,m,t}$, is equal to the sample ready to be shipped to any hospital $h$ $(MSO_{p,m,h,j,t} )$ under transport mode $j$:

17 $$OUTM_{p,m,t} = \mathop \sum \limits_{h,j} MSO_{p,m,h,j,t} ,{ }\forall p,m,t$$

$MSO_{p,m,h,j,t}$ sample $p$ that has left manufacturing facility $m$ arrives at hospital $h$ via transport mode $j$ at time $t$ ($FTR_{p,m,h,j,t}$) after $TT2_{j}$ that is the duration of transport via transport mode $j$:

18 $$FTR_{p,m,h,j,t} = MSO_{{p,m,h,j,t + TT2_{j} }} ,{ }\forall p,m,h,j,t$$

$INH_{p,h,t}$ represents the sample $p$ that arrives at hospital $h$ at time $t$:

19 $$INH_{p,h,t} = \mathop \sum \limits_{m,j} FTD_{p,m,h,j,t} ,{ }\forall p,h,t$$

#### Capacity constraints

 Equation (20) calculates the capacity ($CAP_{m,t}$) of each of the manufacturing facilities $m$ at every time $t$, while Eq. (21) ensures that the therapies in manufacturing do not exceed the available capacity:

20 $$CAP_{m,t} = FCAP_{m} - \mathop \sum \limits_{{p,\hat{t}}} INM_{{p,m,\hat{t}}} ,{ }\forall p,m,t,{ }t - TMFE \le \hat{t} \le t$$

21 $$\mathop \sum \limits_{p} INM_{p,m,t} - \mathop \sum \limits_{p} OUTM_{p,m,t} \le CAP_{m,t} ,{ }\forall p,m,t$$

#### Network structure constraints

 Equation (22)-(23) ensure that matches are established only between existing manufacturing facilities. $E1_{m}$ is a binary variable that takes the value of 1 if a manufacturing facility is established, while it is equal to 0 otherwise. Variables $X1_{c,m}$ and $X2_{m,h}$ are binary variables that can take the value of 1 if only if $E1_{m} = 1$.

22 $$X1_{c,m} \le E1_{m} , \forall c,m$$

23 $$X2_{m,h} \le E1_{m} , \forall c,m$$

Equations (24)-(25) ensure that only one transport mode $j$ for every therapy $p$ at every journey can be selected. Variables $Y1_{p,c,m,j,t}$ and $Y2_{p,m,h,j,t}$ are binary variables that take the value of 1 if a transport mode $j$ is selected for the transportation of therapy $p$ between two facilities at time $t$.

24 $$\mathop \sum \limits_{c,m,j,t} Y1_{p,c,m,j,t} = 1,{ }\forall p,c,m,j,t$$

25 $$\mathop \sum \limits_{m,h,j,t} Y2_{p,m,h,j,t} = 1,{ }\forall p,m,h,j,t$$

Equation (26) adds an upper bound to the total number of manufacturing facilities that can be established.

26 $$\mathop \sum \limits_{m} E1_{m} \le U^{M}$$

#### Demand satisfaction

 The total rate of flow of each therapy $p$ arriving at hospital $h$ must be equal to the corresponding demand (Eq. 27):

27 $$\mathop \sum \limits_{p,h,t} INH_{p,h,t} = NP, \forall p,h,t$$

#### Logical constraints for transportation flows

 Therapies $p$ can be transported from a clinical site $c$ to a manufacturing site $m$ (Eq. 28) and from a manufacturing site $m$ to a hospital $h$ (Eq. 29) if and only if a match between the corresponding facilities has been previously established.

28 $$Y1_{p,c,m,j,t} \le X1_{c,m} ,{ }\forall p,c,m,j,t$$

29 $$Y2_{p,m,h,j,t} \le X2_{m,h} ,{ }\forall p,m,q,j,t$$

Equations (30)-(33) make sure that a match is only made between a leukapheresis site $c$ and its corresponding co-located hospital $h$.

30 $$\mathop \sum \limits_{m,j,t} Y2_{p,m,h1,j,t} \le \mathop \sum \limits_{t} INC_{p,c1,t} \cdot t,{ }\forall p$$

31 $$\mathop \sum \limits_{m,j,t} Y2_{p,m,h2,j,t} \le \mathop \sum \limits_{t} INC_{p,c2,t} \cdot t,{ }\forall p$$

32 $$\mathop \sum \limits_{m,j,t} Y2_{p,m,h3,j,t} \le \mathop \sum \limits_{t} INC_{p,c3,t} \cdot t,{ }\forall p$$

33 $$\mathop \sum \limits_{m,j,t} Y2_{p,m,h4,j,t} \le \mathop \sum \limits_{t} INC_{p,c4,t} \cdot t,{ }\forall p$$

Equations (34)-(37) ensure that a minimum and maximum flow of material exists for a transportation link to be established. The values for $FMIN$ and $FMAX$ can be established by the method presented by Tsiakis et al.^61^. Nonetheless, in this case where every therapy $p$ corresponds to a single patient (material), $FMIN$ and $FMAX$ are assumed to be equal to 0 and 1 respectively.

34 $$LSR_{p,c,m,j,t} \ge Y1_{p,c,m,j,t} \cdot FMIN,{ }\forall p,c,m,j,t$$

35 $$LSR_{p,c,m,j,t} \le Y1_{p,c,m,j,t} \cdot FMAX,{ }\forall p,c,m,j,t$$

36 $$MSOQ_{p,m,hj,t} \ge Y2_{p,m,h,j,t} \cdot FMIN,{ }\forall p,m,h,j,t$$

37 $$MSOQ_{p,m,hj,t} \le Y2_{p,m,h,j,t} \cdot FMAX, \forall p,m,h,j,t$$

#### Time constraints

 Equation (38) calculates the manufacturing time $t$ of therapy $p$ in facility $m$.

38 $$DURM_{p,m,t} = \mathop \sum \limits_{{\hat{t}}} \left( {INM_{{p,m,\hat{t} - 1}} - OUTM_{{p,m,\hat{t}}} } \right) + OUTM_{p,m,t} , \forall p,m,t, \hat{t} \le t$$

Equation (39) establishes the time point when a patient checks into a leukapheresis site $c$, while Eq. (40) presents the time point when therapy $p$ is delivered to hospital $h$.

39 $$CTT_{p} = \mathop \sum \limits_{h,t} INH_{p,h,t} \cdot t, \forall p$$

40 $$STT_{p} = \mathop \sum \limits_{c,t} INC_{p,c,t} \cdot t, \forall p$$

Equation (41) makes sure that the time point a patient checks into a leukapheresis site $c$ chronologically precedes the time point the corresponding therapy $p$ is delivered to the hospital $h$.

41 $$STT_{p} \le CTT_{p} , \forall p$$

Equation (42) ensures that the turnaround time of therapy $p$ is less than or equal to a specified number of days ($ND$).

42 $$TRT_{p} \le ND$$

Equation (43) calculates the total time (vein-to-vein) for a therapy $p \left( {TRT_{p} } \right)$ from the time point that a patient checks into a leukapheresis centre ($STT_{p}$) until the time point that the therapy for this patient is delivered to the hospital location ($CTT_{p}$):

43 $$TRT_{p} = CTT_{p} - STT_{p} , \forall p$$

Equation (44) presents the average return time $\left( {ATRT} \right)$ of all the therapies $p$.

44 $$ATRT = \frac{{\mathop \sum \nolimits_{p} TRT_{p} }}{NP}$$

### Data collection and assumptions

Data regarding costs, demand scales, and current and forward-looking manufacturing facility capacities were obtained through expert discussions with TrakCel Ltd and the Future Targeted Healthcare Manufacturing Hub User Steering Committee. This enabled the development of industrially relevant case studies. For the computational experiments, we consider six candidate manufacturing facilities of three different capacities (4, 10 and 31 parallel production lines). Supplementary Table 3 illustrates the candidate manufacturing facilities as considered in this work, along with their maximum capacities. Their potential locations were placed close to transport hubs, such as airports, ports and train stations. In addition, the locations align with existing cell and gene therapy facilities in Europe, the UK and the US. In this case, we assume that QC is co-located with the manufacturing facility. We consider that final therapies undergo QC in parallel at facilities of approximately 20-fold higher capacity than the manufacturing facility. Therefore, QC capacities are not considered a limiting factor and are not reported here. In addition, the locations of the leukapheresis sites and the hospitals are reported in Supplementary Table 3. Following standard European and UK practices of public and semi-public healthcare systems, we assume that the choice of collaborating hospitals and specialist centres is not under the manufacturer’s sole control. Therefore, these two nodes are considered model inputs and not decision variables. For the leukapheresis sites, we consider a capacity of 8 patients per day, while we assume that capacity is not a bottleneck for the administration of the therapy at the hospital site and thus no upper limit is provided. Following the Advanced Therapy Treatment Centre (ATTC) model as discussed in Papathanasiou et al.^15^, we assume that leukapheresis sites and hospitals are different facilities, located in the same region.

Manufacturing costs are based on the information available from Spink & Steinsapir^64^, whilst QC costs were provided by TrakCel Ltd. as presented in Supplementary Table 1 along with the amortisation period considered for each of the assets. Supplementary Table 2 illustrates the unit transport costs across the various locations and they present estimated costs from a white glove courier with cell therapy processes. We consider facilities of pre-defined capacity and capital investment costs.

Supplementary Table 4 illustrates the durations for all the steps involved in the manufacturing and distribution of CAR T cell therapies. We consider two scenarios, whereby the duration of the manufacturing process is: (a) 7 days which represents a forward-looking scenario of technology developments that can lead to decreased culture times and (b) 19 days, reflecting the current industry average. The time for the QC to be completed is assumed to be 7 days in alignment with a current average duration as provided by expert feedback. A summary of the scenarios considered in this work can be found in Supplementary Table 5. We assume that transportation can happen either within 24 or 48 h, irrespective of the mode of transport (i.e. car, rail etc.). This is to align the model assumptions with standard practice in the cell therapy space where courier contracts are based on the estimated length of delivery and the distance between the candidate locations.

All data generated or analysed during this study are included in this published article [and its supplementary information files]. The model codes developed in this study are available from the corresponding author upon reasonable request.

### Computational statistics

The number of continuous and discrete variables in the model increases with the increased number of patients, reflecting the patient-centric nature of these therapies. This is tracked throughout the model formulation, thereby resulting in a high number of decision variables and constraints that increase the size of the problem. Indicatively, in the case studies considered in this paper, the number of binary variables ranges from 0.62 to 6.18 million, whilst the number of constraints ranges from 2.7 to 26.9 million.

All the models have been implemented in Python 3.7.1 and Pyomo 6.1.2 using the CPLEX 12.9 solver. All computational experiments were performed in a 24-core Xeon E5-2697 machine with 196 GB of RAM. The CPU time ranged between 370 and 3900 s.

## Supplementary Information

Supplementary Information.
