# Supplementary material for: A digital platform for the design of patient-centric supply chains
Source: Sci Rep. 2022 Oct 17;12:17365. doi: 10.1038/s41598-022-21290-5 (PMC9576774; doi:10.1038/s41598-022-21290-5)
Supplement: Supplementary file 1 — Supplementary Information. [file 41598_2022_21290_MOESM1_ESM.pdf]

Supplementary Information

Supplementary Table 1. Manufacturing capacities and costs in USD as considered in this work

	Entity	Value			Amortisation period (years)
Manufacturing Capacity	Number of parallel manufacturing lines (avg)	31	10	4	N/A
Capital Investment Costs	Facility Construction [USD]	1,733,333.33	577,777.78	231,111.11	15
	Validation Expense [USD]	333,333.33	111,111.11	44,444.44	15
	Rent Per Annum [USD/year]	1,600,000	533,333.33	213,333.33	N/A
	Equipment Costs [USD]	2,400,000	800,000	320,000	5
Variable Costs per therapy	QC in house (avg) [USD/therapy]	9312	9312	9312	N/A
	Manufacturing costs (fixed variable) [USD/year]	20,397,000	6,799,000	2,720,000	N/A
	Cost of goods [USD/therapy]	10,500	10,500	10,500	

Supplementary Table 2. Unit transport costs in USD as considered in this work. Costs were provided by TrakCel Ltd

$U1_{c,m,j}$				$U2_{m,h,j}$			
		j_1	j_2			j_1	j_2
c_1	m_1	1040	520	m_1	h_1	1910	910
c_1	m_2	2600	520	m_1	h_2	1910	910
c_1	m_3	2600	520	m_1	h_3	2910	910
c_1	m_4	2900	936	m_1	h_4	1910	910
c_1	m_5	3500	1053	m_2	h_1	2910	910
c_1	m_6	3500	1053	m_2	h_2	400	300
c_2	m_1	2600	520	m_2	h_3	1910	910
c_2	m_2	200	100	m_2	h_4	1910	910
c_2	m_3	2600	520	m_3	h_1	2910	1755
c_2	m_4	2900	936	m_3	h_2	2910	1755
c_2	m_5	3500	1053	m_3	h_3	2910	1755
c_2	m_6	3500	1053	m_3	h_4	2910	1755
c_3	m_1	1840	520	m_4	h_1	2910	1755
c_3	m_2	2000	520	m_4	h_2	2910	1755
c_3	m_3	2900	936	m_4	h_3	2910	1755
c_3	m_4	2900	936	m_4	h_4	2910	1755
c_3	m_5	3500	1053	m_5	h_1	2910	1855
c_3	m_6	3500	1053	m_5	h_2	2910	1855
c_4	m_1	1640	520	m_5	h_3	2910	1855
c_4	m_2	1820	520	m_5	h_4	2910	1755
c_4	m_3	2900	936	m_6	h_1	2910	1855
c_4	m_4	2900	936	m_6	h_2	2910	1855
c_4	m_5	3500	1053	m_6	h_3	2910	1855
c_4	m_6	3500	1053	m_6	h_4	2910	1855

Supplementary Table 3. Candidate manufacturing and QC facilities as considered in this work

	Facility	Location	Capacity (parallel lines)
**Manufacturing sites**	m_1	UK/Stevenage	4
	m_2	EU/Berlin	31
	m_3	EU/Belgium	10
	m_4	USA/Pennsylvania	4
	m_5	USA/Virginia	31
	m_6	UK/Glasgow	10
**Leukapheresis sites**	c_1	London	8 patients/day
	c_2	Glasgow	
	c_3	Manchester	
	c_4	Birmingham	
**Hospitals**	h_1	London	N/A
	h_2	Glasgow	N/A
	h_3	Manchester	N/A
	h_4	Birmingham	N/A

Supplementary Table 4. Processing times as considered in this work

Procedure	Duration (days)
**Leukapheresis**	0.125
**Manufacturing**	7 (assuming future technological developments)<br>19 (current manufacturing time average) [31]
**Quality Control**	7
**Transportation**	Option between 1 and 2 days of transport

Supplementary Table 5. Simulation experiments as designed and assessed in this work

Scenario	Demand (patients/year)	Number of manufacturing sites	Maximum total return time (days)	Time of manufacturing (days)
**1**			Unconstrained	
**2**		2	17	
**3**			18	
**4**			19	7
**5**			Unconstrained	
**6**		Unconstrained	17	
**7**			18	
**8**	200		19	
**9**			Unconstrained	
**10**		2	29	
**11**			30	
**12**			31	19
**13**			Unconstrained	
**14**		Unconstrained	29	
**15**			30	
**16**			31	
**17**			Unconstrained	
**18**			17	
**19**		2	18	
**20**			19	
**21**			Unconstrained	7
**22**			17	
**23**		Unconstrained	18	
**24**			19	
**25**	500		Unconstrained	
**26**			29	
**27**		2	30	
**28**			31	
**29**			Unconstrained	19
**30**			29	
**31**		Unconstrained	30	
**32**			31	
**33**			Unconstrained	
**34**		2	17	
**35**			18	
**36**			19	7
**37**			Unconstrained	
**38**		Unconstrained	17	
**39**			18	
**40**	1000		19	
**41**			Unconstrained	
**42**		3	29	
**43**		2	30	
**44**			31	19
**45**			Unconstrained	
**46**		Unconstrained	29	
**47**			30	

**48**			31	
**49**	2000	2	Unconstrained	7
**50**			17	
**51**			18	
**52**			19	
**53**		Unconstrained	Unconstrained	
**54**			17	
**55**			18	
**56**			19	

Supplementary Table 6. Model nomenclature for the Mixed Integer Linear Programming supply chain optimisation problem formulation

INDICES

$\boldsymbol{c}$	leukapheresis sites
$\boldsymbol{h}$	hospitals
$\boldsymbol{j}$	transport modes
$\boldsymbol{m}$	manufacturing sites
$\boldsymbol{p}$	Patients/therapies
$\boldsymbol{t}$	time periods

PARAMETERS

$\boldsymbol{CIM_m}$	Capital investment for manufacturing facility m	USD
$\boldsymbol{CFVM_m}$	Fixed variable manufacturing cost for manufacturing facility m (personnel, facilities and equipment maintenance costs)	USD
$\boldsymbol{CVM_p}$	Variable cost of materials required for therapy p	USD
$\boldsymbol{CQC}$	Quality control cost when in house	USD therapy^{-1}
$\boldsymbol{FCAP_m}$	Total capacity of manufacturing site m	therapies
$\boldsymbol{FMAX}$	Maximum flow	-
$\boldsymbol{FMIN}$	Minimum flow	-
$\boldsymbol{INC_{p,c,t}}$	Patient p arriving for leukapheresis site c at time t	patients
$\boldsymbol{TLS}$	Duration of leukapheresis procedure	days

$\boldsymbol{TMFE}$	Duration of the manufacturing process	days
$\boldsymbol{TQC}$	Duration of Quality Control	days
$\boldsymbol{NT}$	Number of time periods	-
$\boldsymbol{NP}$	Number of patients/therapies	-
$\boldsymbol{TT1_j}$	Transport time from leukapheresis site c to manufacturing facility m using transport mode j	days
$\boldsymbol{TT2_j}$	Transport time from manufacturing facility m to hospital h using transport mode j	days
$\boldsymbol{U1_{c,m,j}}$	Unit transport cost from leukapheresis site c to manufacturing facility m using transport mode j	USD therapy^{-1} day^{-1}
$\boldsymbol{U2_{m,h,j}}$	Unit transport cost from manufacturing facility m to hospital h using transport mode j	USD therapy^{-1} day^{-1}
**BINARY VARIABLES**		
$\boldsymbol{E1_m}$	1 if manufacturing facility m is established	-
$\boldsymbol{X1_{c,m}}$	1 if a match between a leukapheresis site c and manufacturing facility m is established	-
$\boldsymbol{X2_{m,h}}$	1 if a match between manufacturing facility m and a hospital h is established	-
$\boldsymbol{Y1_{p,c,m,j,t}}$	1 a sample p is transferred from a leukapheresis site c to a manufacturing facility m via mode j at time t	-

$\boldsymbol{Y2_{p,m,h,j,t}}$	1 a sample p is transferred from a manufacturing facility m to a hospital h via mode j at time t	-
**VARIABLES**		
$\boldsymbol{ATRT}$	Average return time	days
$\boldsymbol{CAP_{m,t}}$	Capacity of manufacturing facility m at time t	therapies day^{-1}
$\boldsymbol{CTM_p}$	Total manufacturing cost of therapy p	USD therapy^{-1}
$\boldsymbol{CTT_p}$	Completion time of treatment for patient p	-
$\boldsymbol{DURM_{p,m,t}}$	1 only for the time points t at which a therapy p is manufactured in facility m	-
$\boldsymbol{FTD_{p,m,h,j,t}}$	Final therapy that left from manufacturing facility m arriving at hospital h via mode j at time t	-
$\boldsymbol{FTR_{p,m,h,j,t}}$	Therapy p leaving manufacturing facility m to hospital h via mode j at time t	-
$\boldsymbol{INH_{p,h,t}}$	Therapy p arriving at hospital h at time t	-
$\boldsymbol{INM_{p,m,t}}$	Therapy p arriving at manufacturing facility m at time t	-
$\boldsymbol{LSA_{p,c,m,j,t}}$	Therapy p that left leukapheresis site c and arrived at manufacturing facility m via mode j at time t	-
$\boldsymbol{LSR_{p,c,m,j,t}}$	Therapy p that left leukapheresis site c to go to manufacturing facility m via mode j at time t	-
$\boldsymbol{MSO_{p,m,h,j,t}}$	Therapy p leaving manufacturing facility m to go to hospital h via mode j at time t	-

$\boldsymbol{OUTC_{p,c,t}}$	Therapy p leaving leukapheresis site c at time t	-
$\boldsymbol{OUTM_{p,m,t}}$	Therapy p leaving manufacturing facility m at time t	-
$\boldsymbol{RATIO_{m,t}}$	Percentage of utilisation of manufacturing site m at time t	-
$\boldsymbol{STT_p}$	Starting time of treatment for patient p	-
$\boldsymbol{TOTCOST}$	Total supply chain cost	USD
$\boldsymbol{TRT_p}$	Total return time of therapy	days
$\boldsymbol{TTC_p}$	Total transport cost per therapy p	USD therapy^{-1}

Supplementary Table 7. Model nomenclature for the Mixed Integer Linear Programming demand distribution problem formulation

INDICES

c	Leukapheresis sites	
p	Patients	
t	Time	
**PARAMETERS**		
D	Total quarterly demand	Patients
$MLCAP_c$	Maximum capacity of leukapheresis centre c	Patients
TLS	Duration of leukapheresis procedure	Days
**VARIABLES**		
$INC_{p,c,t}$	Patient p arriving for leukapheresis site c at time t	Patients

Supplementary Table 8. Demand scenarios as considered in this work

ANNUAL DEMAND (PATIENTS)	QUARTERLY DEMAND (D) (PATIENTS)
**200**	50
**500**	125
**1000**	248
**2000**	495

Supplementary Table 9. Mixed Integer Linear Programming demand distribution problem formulation

EQUATION	DESCRIPTION	MATHEMATICAL FORMULATION
**ALLOCATION CONSTRAINT**	Every patient p is allocated to a leukapheresis centre c only one time inside a given time horizon t	$\sum_{c,t} INC_{p,c,t} = 1 \; \forall \, p$
**DEMAND**	The number of patient p that start leukapheresis treatment inside a given time horizon t must be equal to the total therapy demand D. The values for the demand are based on the simulation scenario and are illustrated in Table S4	$\sum_{p,c,t} INC_{p,c,t} = D$
**CAPACITY OF LEUKAPHERE SIS CENTRE**	The capacity $CAPL_{c,t}$ of each leukapheresis centre c is calculated for every time point t as the difference between the maximum capacity $MLCAP_c$ and the total patients $\sum_p INC_{p,c,t}$ arriving at centre c at time t	$CAPL_{c,t} = MLCAP_c - \sum_p INC_{p,c,t} \; \forall \, c, t$
**CAPACITY CONSTRAINT**	Constraint ensuring that leukapheresis centres c do not exceed capacity	$\sum_p INC_{p,c,t} \leq CAPL_{c,t} \;\; \forall \, l, t$

**OBJECTIVE FUNCTION**	For the objective function a dummy variable $DIFF$ is used and its value is maximised, therefore driving the model to allocate every patient to a time slot within the time horizon $T = 90\ days$	$DIFF - \sum_{p,c,t} INC_{p,c,t} = 0$

The leukapheresis procedure lasts 2 to 3 hours on average therefore $MLCAP_c$ that is the maximum capacity of a leukapheresis site c and is equal to 8 patients/day.

Supplementary Table 10. Results for each scenario

Scenario	Average return time (days)	Average cost per therapy (USD)	Average manufacturing cost per therapy (USD)	Average quality control cost per therapy (USD)	Average transport cost per therapy (USD)	Manufacturing facilities	Transport modes
**1**	19	141775	120308	19788	1679	m_1, m_4	j_1, j_2
**2**	17	144383	120308	19788	4287	m_1, m_4	j_1
**3**	18	142736	120308	19788	2640	m_1, m_4	j_1, j_2
**4**	19	141775	120308	19788	1679	m_1, m_4	j_1, j_2
**5**	19	141775	120308	19788	1679	m_1, m_4	j_1, j_2
**6**	17	144383	120308	19788	4287	m_1, m_4	j_1
**7**	18	142736	120308	19788	2640	m_1, m_4	j_1, j_2
**8**	19	141775	120308	19788	1679	m_1, m_4	j_1, j_2
**9**	31	323121	300770	19788	2564	m_3, m_6	j_2
**10**	29	326356	300770	19788	5798	m_3, m_6	j_1
**11**	30	324258	300770	19788	3700	m_3, m_6	j_1, j_2
**12**	31	323121	300770	19788	2564	m_3, m_6	j_2
**13**	31	292559	270693	19788	2079	m_1, m_3, m_4	j_2
**14**	29	295378	270693	19788	4898	m_1, m_3, m_4	j_1
**15**	30	293654	270693	19788	3173	m_1, m_3, m_4	j_1, j_2
**16**	31	292559	270693	19788	2079	m_1, m_3, m_4	j_2
**17**	19	142605	120308	19788	2509	m_3, m_6	j_2
**18**	17	145836	120308	19788	5740	m_3, m_6	j_1
**19**	18	143745	120308	19788	3649	m_3, m_6	j_1, j_2
**20**	19	142605	120308	19788	2509	m_3, m_6	j_2
**21**	19	130073	108277	19788	2008	m_1, m_3, m_4	j_2
**22**	17	132818	108277	19788	4753	m_1, m_3, m_4	j_1
**23**	18	131127	108277	19788	3062	m_1, m_3, m_4	j_1, j_2
**24**	19	130073	108277	19788	2008	m_1, m_3, m_4	j_2
**25**	31	201441	180462	19788	1191	m_2	j_2
**26**	29	203807	180462	19788	3557	m_2	j_1
**27**	30	202520	180462	19788	2270	m_2	j_1, j_2
**28**	31	201441	180462	19788	1191	m_2	j_2
**29**	31	201441	180462	19788	1191	m_2	j_2
**30**	29	203807	180462	19788	3557	m_2	j_1
**31**	30	202520	180462	19788	2270	m_2	j_1, j_2
**32**	31	201441	180462	19788	1191	m_2	j_2
**33**	19	111944	90958	19788	1197	m_2	j_2
**34**	17	114261	90958	19788	3514	m_2	j_1
**35**	18	112990	90958	19788	2244	m_2	j_1, j_2
**36**	19	111944	90958	19788	1197	m_2	j_2
**37**	19	106975	84895	19788	2292	m_1, m_3, m_4, m_6	j_1, j_2
**38**	17	114261	90958	19788	3514	m_2	j_1
**39**	18	108053	84895	19788	3370	m_1, m_3, m_4, m_6	j_1, j_2
**40**	19	106975	84895	19788	2292	m_1, m_3, m_4, m_6	j_1, j_2
**41**	31	203466	181917	19788	1761	m_2, m_5	j_1, j_2
**42**	29	217773	194045	19788	3940	m_1, m_2, m_5	j_1

**43**	30	204349	181917	19788	2644	m_2, m_5	j_1, j_2
**44**	31	203466	181917	19788	1761	m_2, m_5	j_1, j_2
**45**	31	197260	175853	19788	1619	m_1, m_2, m_3, m_4, m_6	j_1, j_2
**46**	29	217773	194045	19788	3940	m_1, m_2, m_5	j_1
**47**	30	198065	175853	19788	2424	m_1, m_2, m_3, m_4, m_6	j_1, j_2
**48**	31	197260	175853	19788	1619	m_1, m_2, m_3, m_4, m_6	j_1, j_2
**49**	19	112384	91142	19788	1454	m_2, m_5	j_1, j_2
**50**	17	114657	91142	19788	3727	m_2, m_5	j_1
**51**	18	113261	91142	19788	2331	m_2, m_5	j_1, j_2
**52**	19	112384	91142	19788	1454	m_2, m_5	j_1, j_2
**53**	19	93985	72914	19788	1283	m_1, m_2, m_3, m_4	j_1, j_2
**54**	17	105100	82028	19788	3284	m_1, m_2, m_3, m_6	j_1
**55**	18	94781	72914	19788	2080	m_1, m_2, m_3, m_4	j_1, j_2
**56**	19	93985	72914	19788	1283	m_1, m_2, m_3, m_4	j_1, j_2

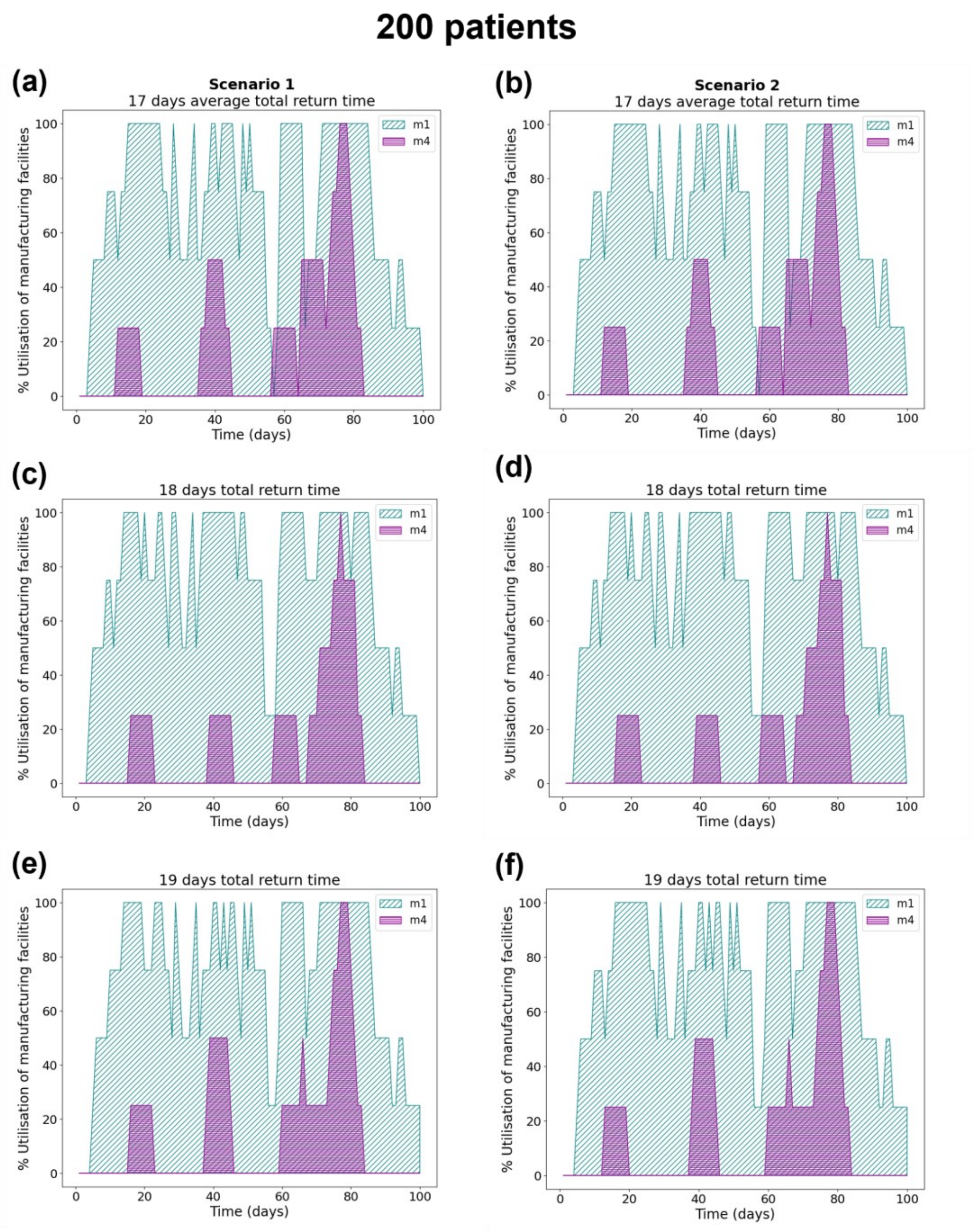

Supplementary Figure 1. Utilisation of manufacturing facilities built for 7 days manufacturing duration for 200 patients/year for 17, 18 or 19 days average total return time. Scenario 1 and Scenario 2 correspond to: unconstrained number of manufacturing and constrained number of manufacturing facilities respectively.

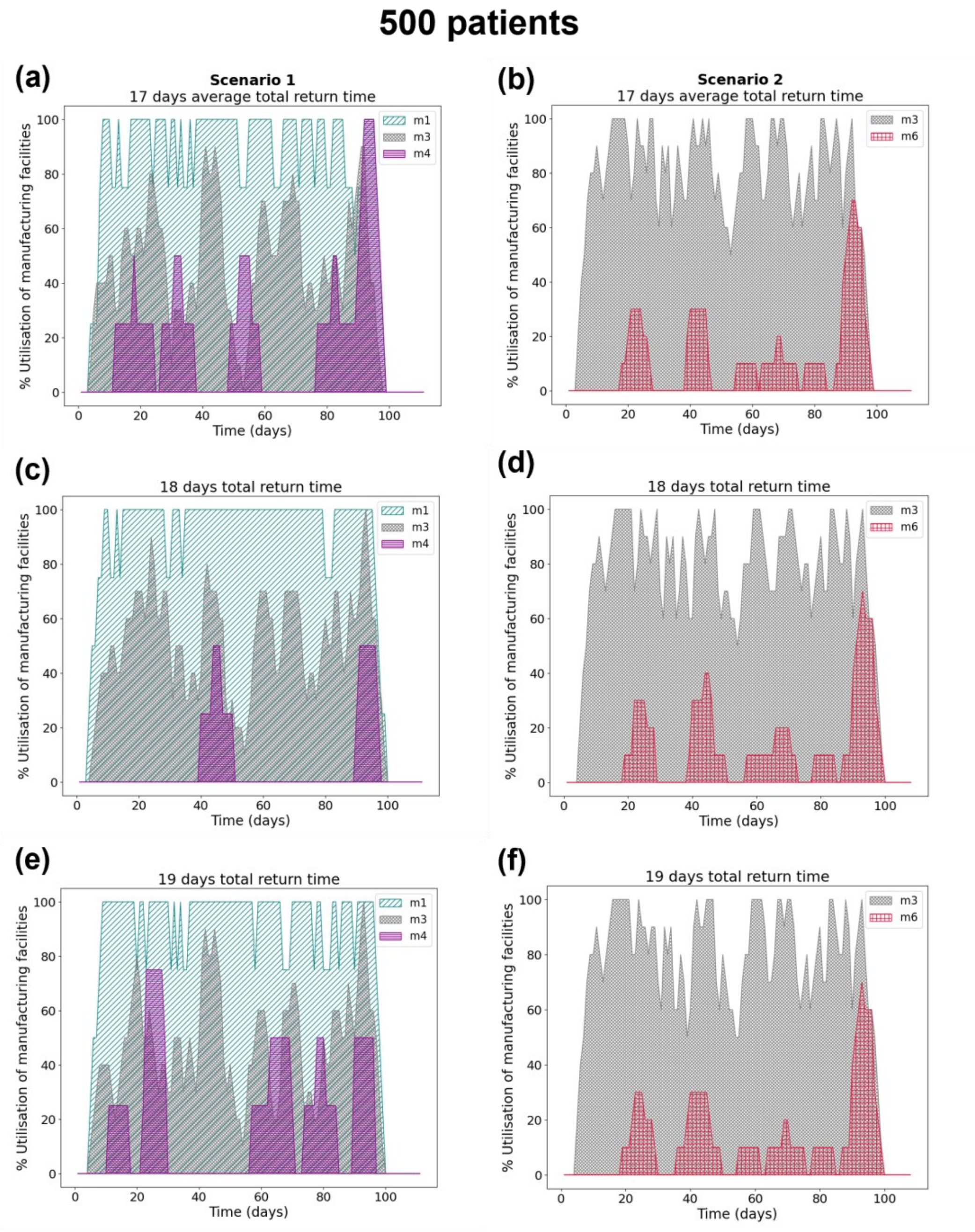

Supplementary Figure 2. Utilisation of manufacturing facilities built for 7 days manufacturing duration for 500 patients/year for 17, 18 or 19 days average total return time. Scenario 1 and Scenario 2 correspond to: unconstrained number of manufacturing and constrained number of manufacturing facilities respectively.

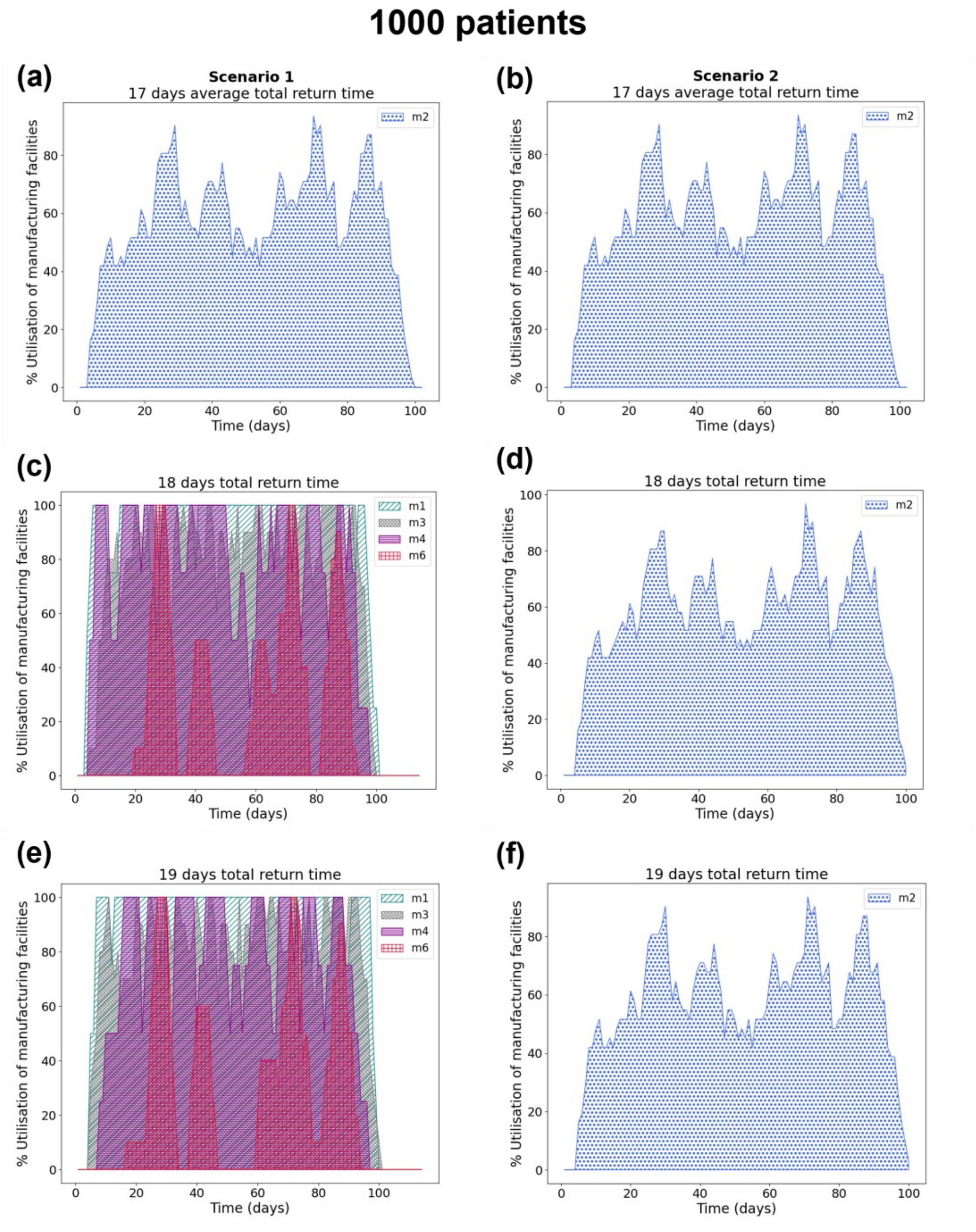

Supplementary Figure 3. Utilisation of manufacturing facilities built for 7 days manufacturing duration for 1000 patients/year for 17, 18 or 19 days average total return time. Scenario 1 and Scenario 2 correspond to: unconstrained number of manufacturing and constrained number of manufacturing facilities respectively.

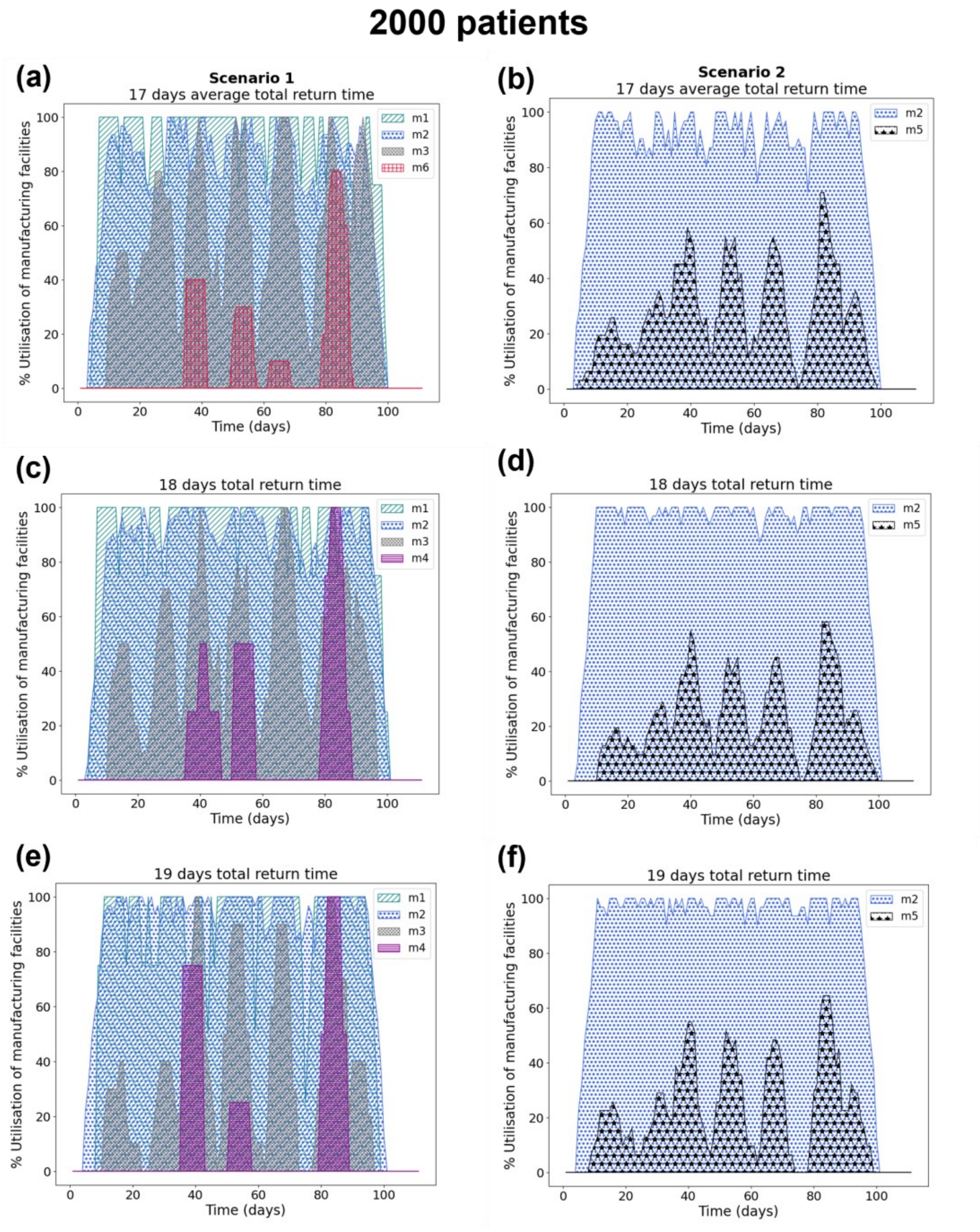

Supplementary Figure 4. Utilisation of manufacturing facilities built for 7 days manufacturing duration for 2000 patients/year for 17, 18 or 19 days average total return time. Scenario 1 and Scenario 2 correspond to: unconstrained number of manufacturing and constrained number of manufacturing facilities respectively.

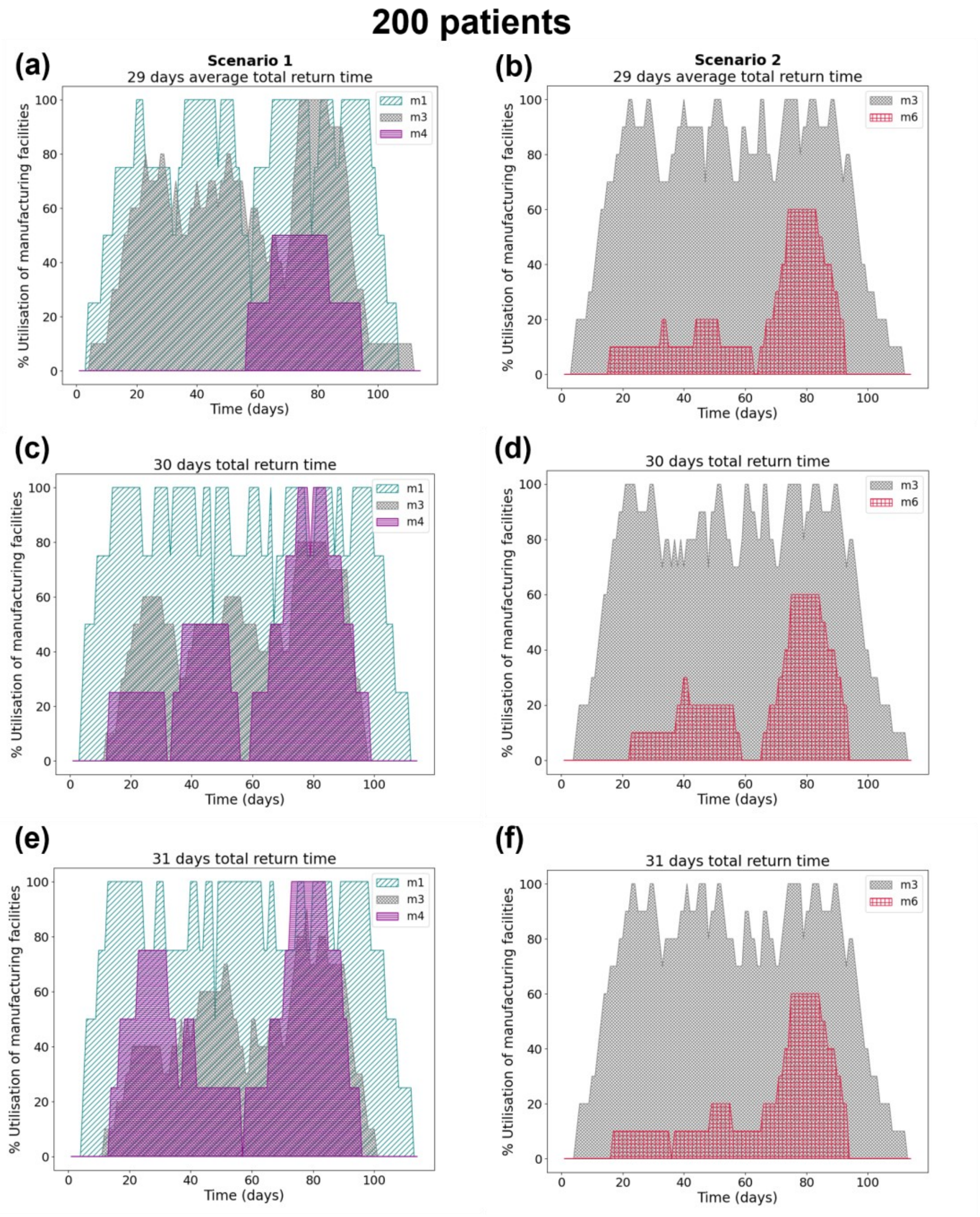

Supplementary Figure 5. Utilisation of manufacturing facilities built for 19 days manufacturing duration for 200 patients/year for 29, 30 or 31 days average total return time. Scenario 1 and Scenario 2 correspond to: unconstrained number of manufacturing and constrained number of manufacturing facilities respectively.

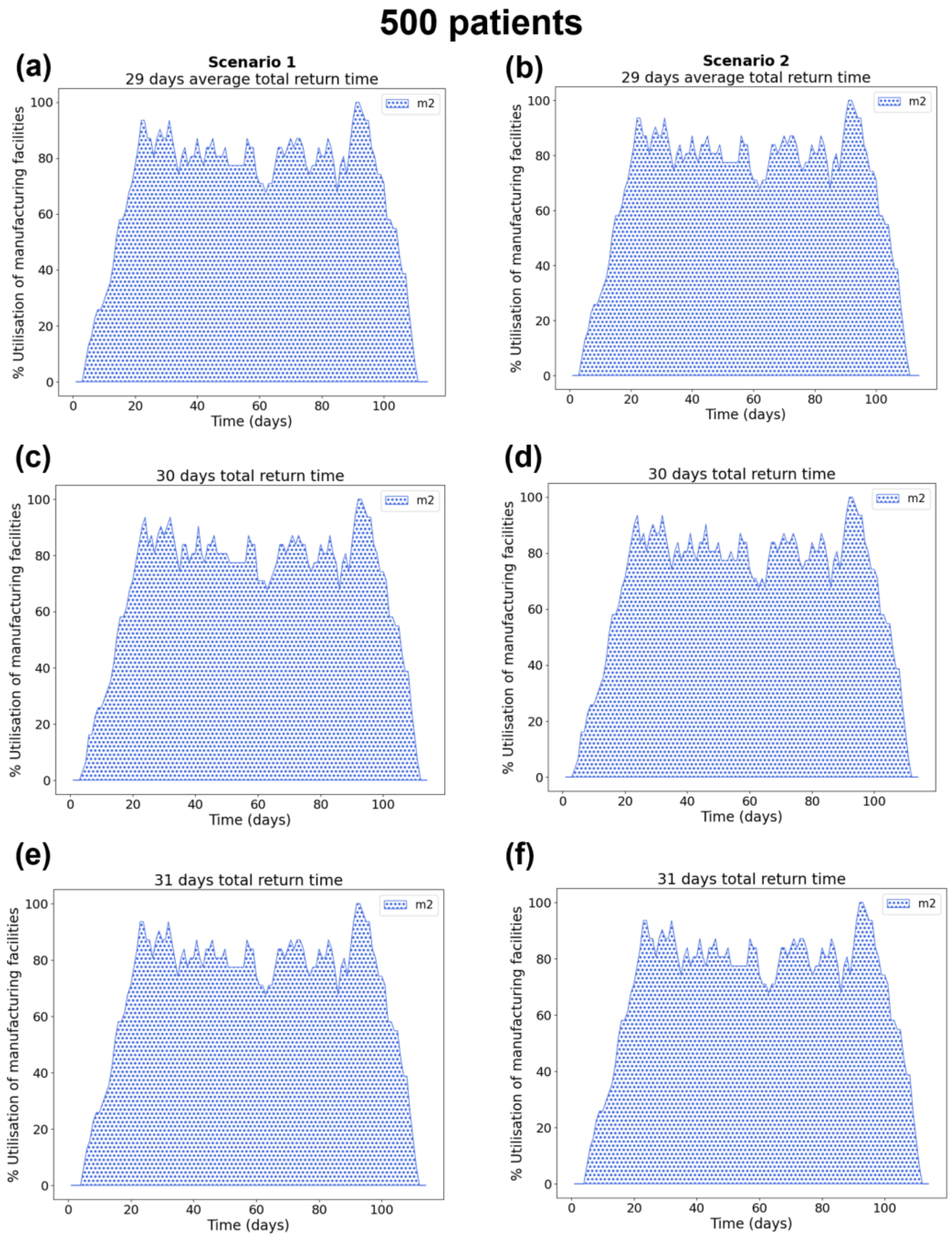

Supplementary Figure 6. Utilisation of manufacturing facilities built for 19 days manufacturing duration for 500 patients/year for 29, 30 or 31 days average total return time. Scenario 1 and Scenario 2 correspond to: unconstrained number of manufacturing and constrained number of manufacturing facilities respectively.

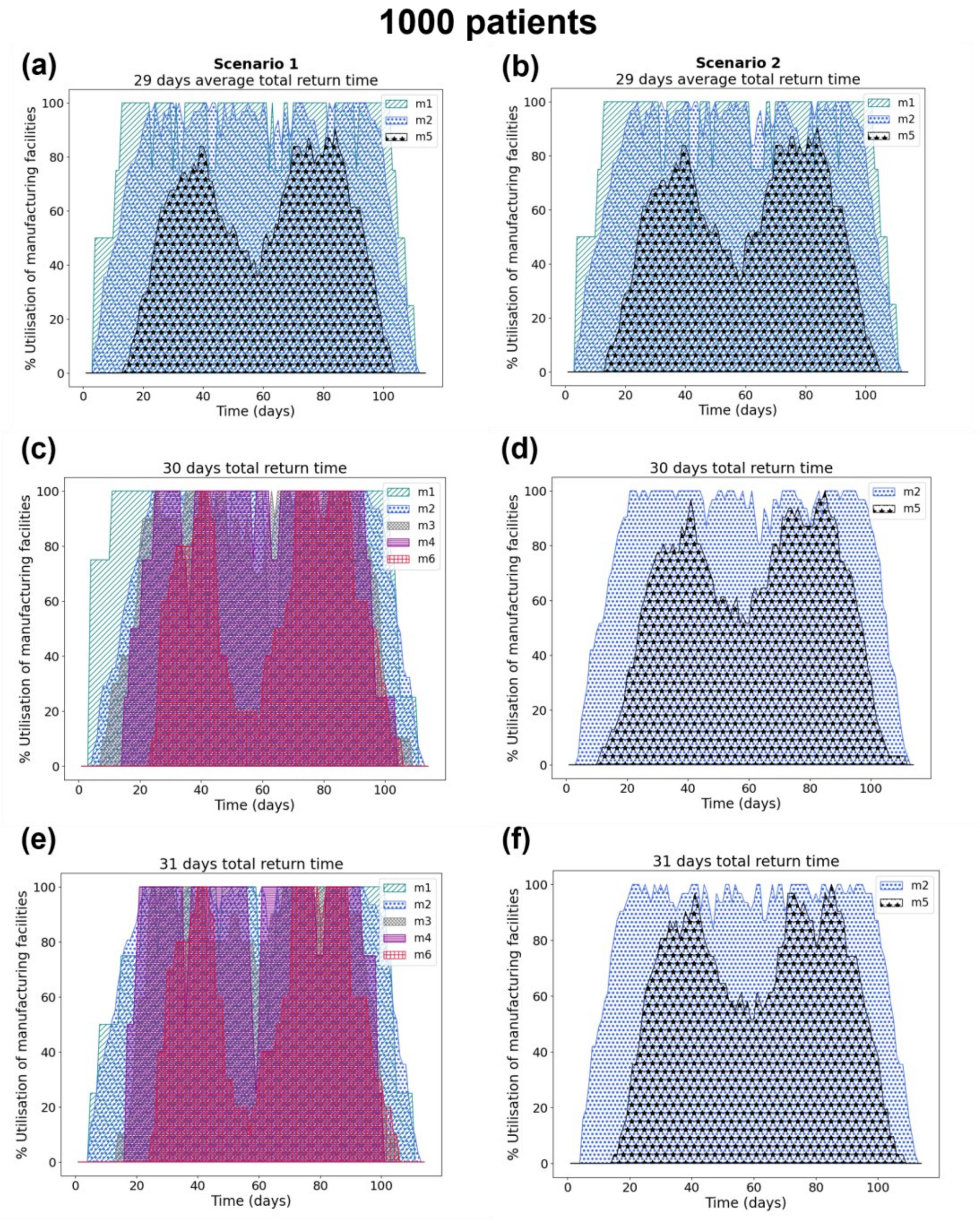

Supplementary Figure 7. Utilisation of manufacturing facilities built for 19 days manufacturing duration for 1000 patients/year for 29, 30 or 31 days average total return time. Scenario 1 and Scenario 2 correspond to: unconstrained number of manufacturing and constrained number of manufacturing facilities respectively.

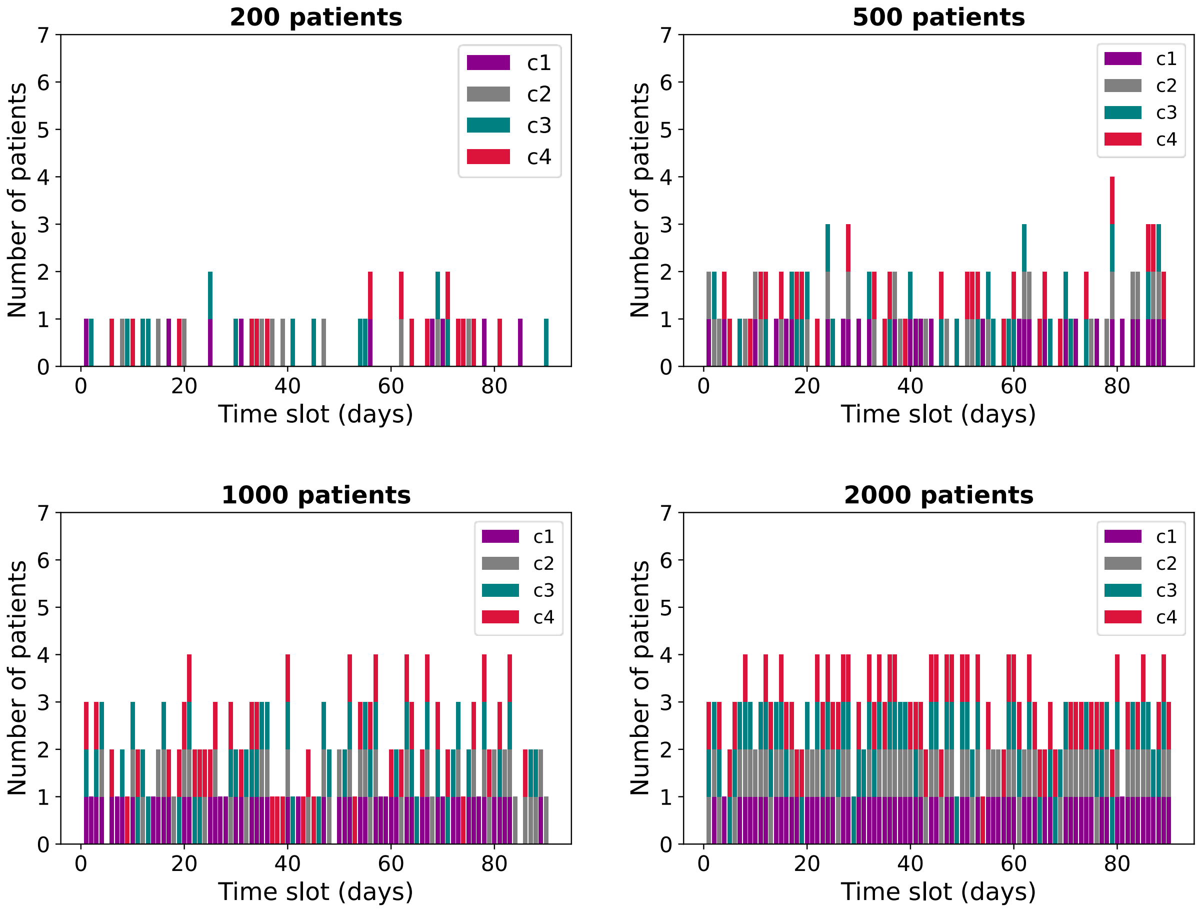

Supplementary Figure 8. Randomised demand profiles for incoming patients to leukapheresis centres (c1, c2, c3, c4) used in each scenario, generated with the algorithm presented in Supplementary Table 9.
